# Supplementary material for: Inhibition of CMP-sialic acid transport by endogenous 5-methyl CMP
Source: PLoS One. 2021 Jun 3;16(6):e0249905. doi: 10.1371/journal.pone.0249905 (PMC8174729; doi:10.1371/journal.pone.0249905)
Supplement: S1 Table — (DOCX) [file pone.0249905.s001.docx]

**S1 Table.** **Data collection and refinement statistics**

| **Data collection** |  |
| --- | --- |
| Space group | C2 |
| Crystals (#) | 2 |
| Cell dimensions (Å) |  |
| *a* | 49.83 |
| *b* | 49.40 |
| *c* | 137.63 |
| α, γ = 90; β = (°) | 92.57 |
| Resolution (Å) | 48 – 1.80  (1.86-1.80) |
| *R*_merge_ | 0.13 (2.37) |
| *R*_pim_ | 0.06 (1.08) |
| *I* / σ*I* | 7.0 (0.7) |
| CC_1/2_ in outer shell | 0.21 |
| Completeness (%) | 100 (99.9) |
| Redundancy | 6.6 (5.6) |
|  |  |
|  |  |
| **Refinement** |  |
| Resolution (Å) | 48 – 1.80  (1.85 – 1.80) |
| No. reflections  No. in free set | 31207  1582 |
| *R*_work_ / *R*_free_ | 18.0/19.8  (37.3/38.4) |
| No. atoms |  |
| Protein | 2485 |
| Ligands | 194 |
| Water | 162 |
| *B*-factors |  |
| Protein | 40.5 |
| Ligand | 64.9 |
| Water | 51.1 |
| R.m.s. deviations |  |
| Bond lengths (Å) | 0.006 |
| Bond angles (°) | 0.736 |
|  |  |

*Values in parentheses are for the highest-resolution shell.
