# Supplementary figures and images for: Inhibition of CMP-sialic acid transport by endogenous 5-methyl CMP
Source: PLoS One. 2021 Jun 3;16(6):e0249905. doi: 10.1371/journal.pone.0249905 (PMC8174729; doi:10.1371/journal.pone.0249905)

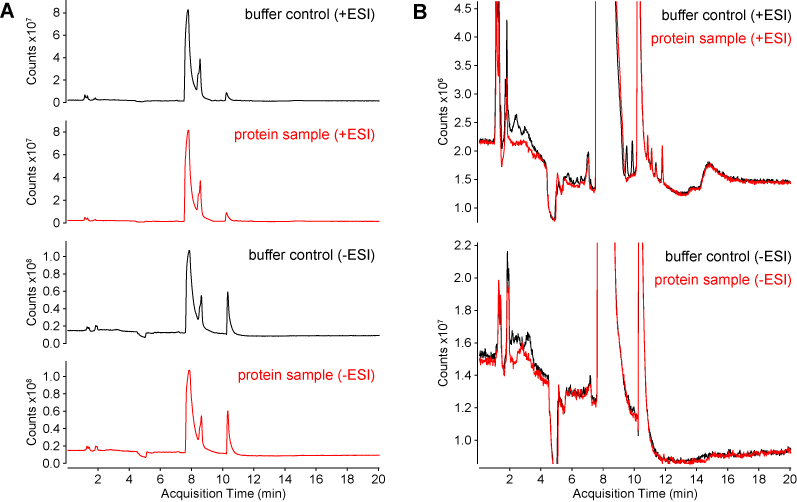

Supplement: S1 Fig — Phenol-chloroform extractions were performed on either a buffer-only control (black traces) or a sample containing purified CST protein (red traces). The aqueous layers were run on an LC-MS system in either positive or negative ESI mode with the resulting total ion current chromatograms shown in panel A. The chromatograms for each ESI mode are overlaid for better comparison in panel B. (TIF) [file pone.0249905.s002.tif]

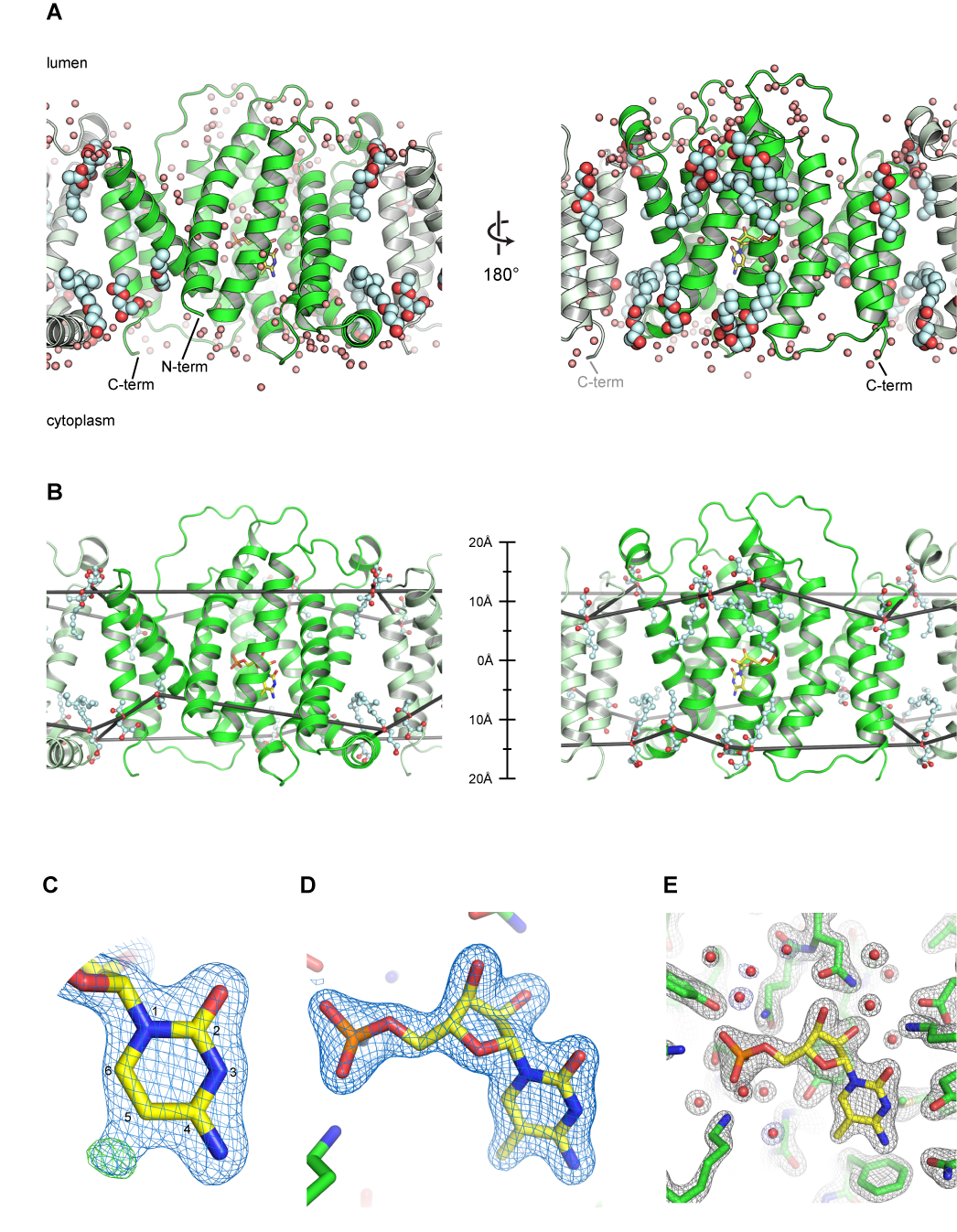

Supplement: S2 Fig — A) Two views (front and back) of the CST-m5CMP structure are shown, with the lumenal and cytoplasmic sides of the protein indicated. Water molecules are shown as small pink spheres and monoolein lipid molecules are shown as larger spheres with red indicating oxygen and light cyan indicating carbon. The CST molecule in the asymmetric unit is shown as a green cartoon. Symmetry-related CST molecules to the left and right are shown (as light green cartoons) since some of the lipids and waters mediate crystal contacts between CST molecules. B) The same views of the CST-m5CMP structure that are shown in panel A are show here as well, except only the monoolein lipid molecules are shown in ball-and-stick representation. The oxygen atoms of the acyl-ester linkages of adjacent monooleins are connected by a black line to give a first order approximation of the shape and thickness of a lipid bilayer that would interact with CST. A scale bar is shown to indicate the extent of the protein-lipid interface on either side of the protein. C) 2Fo-Fc (blue mesh, 1.5σ) and Fo-Fc (green mesh, 3.5σ) electron density maps are shown. These maps were obtained by using molecular replacement to solve the structure of the CST-m5CMP crystal, using the CST-CMP structure as a search model. The cytosine group of CMP from the search model is shown as yellow sticks. The atoms of the pyrimidine ring are numbered and density for a methyl group at the C-5 position is clearly observed in both maps. D) Simulated-annealing omit map of the CST-m5CMP structure, calculated without m5CMP modeled. Density is shown for the m5CMP molecule, contoured at 1.5σ. The m5CMP model is shown for reference. E) A 2Fo-Fc map of the final refined structure of the CST-m5CMP structure is shown. The gray mesh is contoured at 1.8σ and the blue mesh is contoured at 1σ to show the weaker density for some of the waters. (TIF) [file pone.0249905.s003.tif]

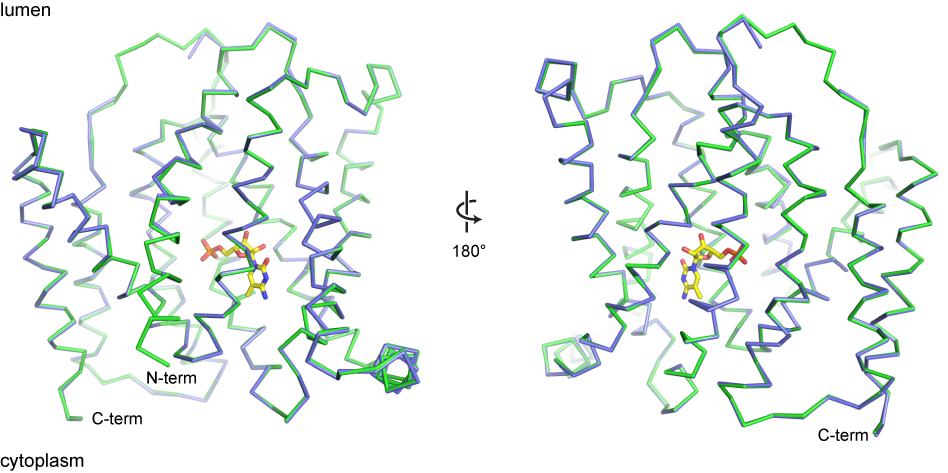

Supplement: S3 Fig — The CST-CMP (blue) and CST-m5CMP (green) structures are shown as Cα traces and are superimposed to show the high structural identity. Front and back views are shown with the lumenal and cytoplasmic sides of the protein indicated. m5CMP is shown as yellow sticks. (TIF) [file pone.0249905.s004.tif]

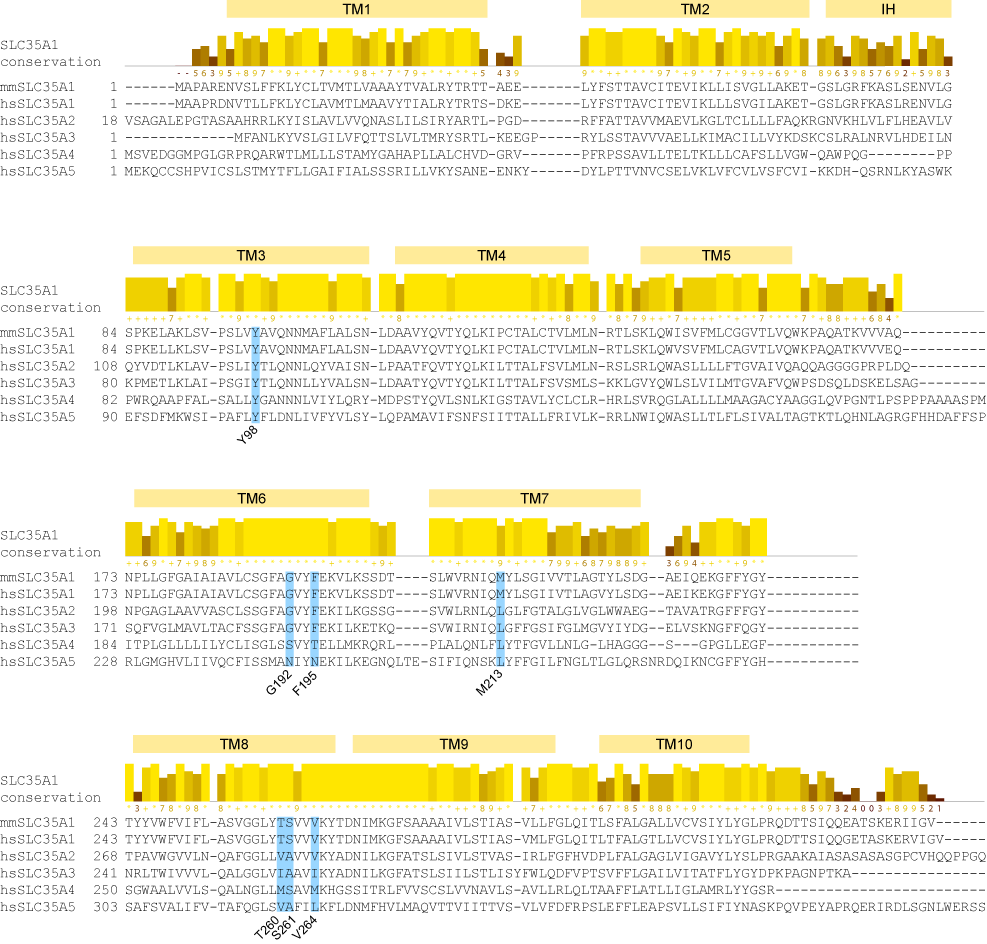

Supplement: S4 Fig — A sequence alignment between mouse CST (mmSLC35A1), human CST (hsSLC35A1), and other human SLC35A family members is shown. The bar graph above the alignment shows the sequence conservation among 126 SLC35A1 orthologs. The row of numbers and symbols under the bar graph indicates the degree of conservation, with a higher number indicating greater conservation, a “+” symbol indicating near-complete identity, and an “*” symbol indicating complete identity. Residues relevant to m5CMP interactions that are discussed in the text are highlighted and labeled. (TIF) [file pone.0249905.s005.tif]

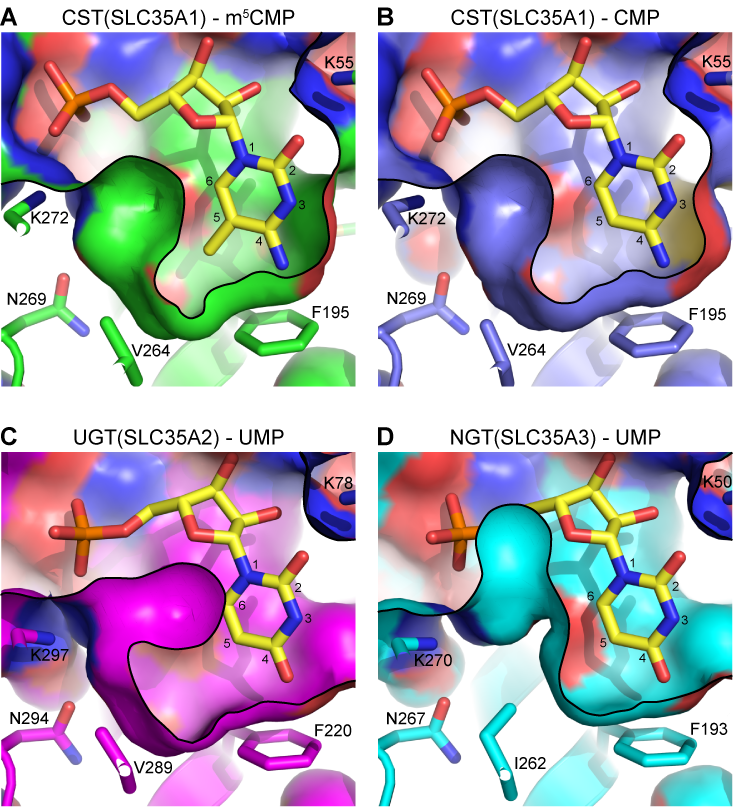

Supplement: S5 Fig — A and B) The depiction of the hydrophobic pocket in CST is reproduced from Fig 5 for comparison. C and D) Structural homology models of UGT (SLC35A2, panel C) and NGT (SLC35A3, panel D), which respectively have 44% and 41% sequence identity with the human protein, are shown with UMP docked in their substrate binding sites. The models were superimposed on the CST-m5CMP structure and the same view of a slice through a surface representation of the substrate-binding cavity is shown. In all panels, key residues are labeled and the atoms for the pyrimidine ring are numbered. (TIF) [file pone.0249905.s006.tif]
