# Supplementary material for: Inhibition of CMP-sialic acid transport by endogenous 5-methyl CMP
Source: PLoS One. 2021 Jun 3;16(6):e0249905. doi: 10.1371/journal.pone.0249905 (PMC8174729; doi:10.1371/journal.pone.0249905)
Supplement: S1 File — (PDF) [file pone.0249905.s007.pdf]

Full wwPDB X-ray Structure Validation Report (i)

Jun 8, 2020 – 02:19 PM EDT

PDB ID	:	6XBO
Title	:	X-ray crystal structure of the mouse CMP-Sialic acid transporter in complex with 5-methyl CMP
Deposited on	:	2020-06-06
Resolution	:	1.80 Å(reported)

This is a Full wwPDB X-ray Structure Validation Report.

This report is produced by the wwPDB biocuration pipeline after annotation of the structure.

We welcome your comments at *validation@mail.wwpdb.org*
A user guide is available at
https://www.wwpdb.org/validation/2017/XrayValidationReportHelp
with specific help available everywhere you see the (i) symbol.

The following versions of software and data (see references (i)) were used in the production of this report:

MolProbity	:	4.02b-467
Mogul	:	1.8.5 (274361), CSD as541be (2020)
Xtriage (Phenix)	:	1.13
EDS	:	2.12
buster-report	:	1.1.7 (2018)
Percentile statistics	:	20191225.v01 (using entries in the PDB archive December 25th 2019)
Refmac	:	5.8.0158
CCP4	:	7.0.044 (Gargrove)
Ideal geometry (proteins)	:	Engh & Huber (2001)
Ideal geometry (DNA, RNA)	:	Parkinson et al. (1996)
Validation Pipeline (wwPDB-VP)	:	2.12

CONFIDENTIAL VALIDATION REPORT

1 Overall quality at a glance (i)

The following experimental techniques were used to determine the structure:
X-RAY DIFFRACTION

The reported resolution of this entry is 1.80 Å.

Percentile scores (ranging between 0-100) for global validation metrics of the entry are shown in the following graphic. The table shows the number of entries on which the scores are based.

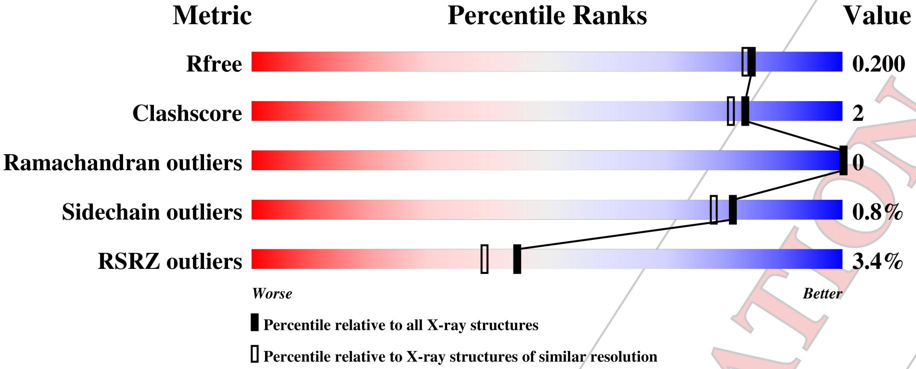

Metric	Whole archive (#Entries)	Similar resolution (#Entries, resolution range(Å))
R_{free}	130704	5950 (1.80-1.80)
Clashscore	141614	6793 (1.80-1.80)
Ramachandran outliers	138981	6697 (1.80-1.80)
Sidechain outliers	138945	6696 (1.80-1.80)
RSRZ outliers	127900	5850 (1.80-1.80)

The table below summarises the geometric issues observed across the polymeric chains and their fit to the electron density. The red, orange, yellow and green segments on the lower bar indicate the fraction of residues that contain outliers for >=3, 2, 1 and 0 types of geometric quality criteria respectively. A grey segment represents the fraction of residues that are not modelled. The numeric value for each fraction is indicated below the corresponding segment, with a dot representing fractions <=5% The upper red bar (where present) indicates the fraction of residues that have poor fit to the electron density. The numeric value is given above the bar.

Mol	Chain	Length	Quality of chain
1	A	330	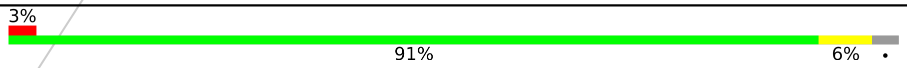

CONFIDENTIAL VALIDATION REPORT

2 Entry composition (i)

There are 5 unique types of molecules in this entry. The entry contains 2841 atoms, of which 0 are hydrogens and 0 are deuteriums.

In the tables below, the ZeroOcc column contains the number of atoms modelled with zero occupancy, the AltConf column contains the number of residues with at least one atom in alternate conformation and the Trace column contains the number of residues modelled with at most 2 atoms.

- Molecule 1 is a protein called CMP-sialic acid transporter.

Mol	Chain	Residues	Atoms					ZeroOcc	AltConf	Trace
			Total	C	N	O	S			
1	A	319	2463	1621	381	446	15	0	10	0

There are 8 discrepancies between the modelled and reference sequences:

Chain	Residue	Modelled	Actual	Comment	Reference
A	323	ASN	-	expression tag	UNP Q61420
A	324	SER	-	expression tag	UNP Q61420
A	325	LEU	-	expression tag	UNP Q61420
A	326	GLU	-	expression tag	UNP Q61420
A	327	VAL	-	expression tag	UNP Q61420
A	328	LEU	-	expression tag	UNP Q61420
A	329	PHE	-	expression tag	UNP Q61420
A	330	GLN	-	expression tag	UNP Q61420

- Molecule 2 is 5-METHYLCYTIDINE-5'-MONOPHOSPHATE (three-letter code: 5MC) (formula: $C_{10}H_{16}N_3O_8P$) (labeled as "Ligand of Interest" by author).

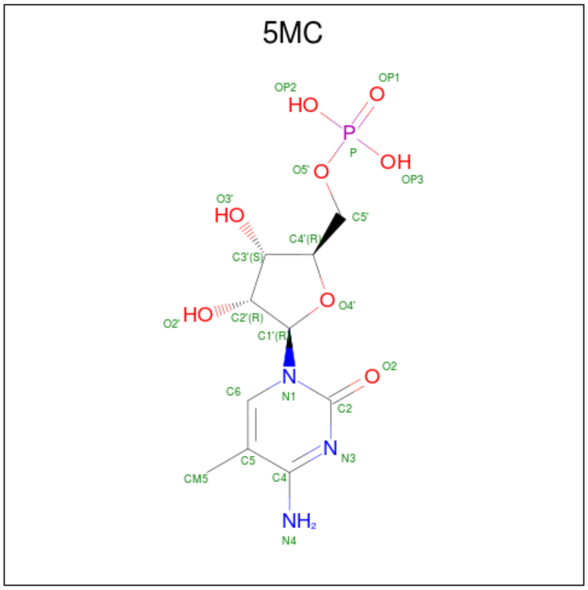

CONFIDENTIAL VALIDATION REPORT

Mol	Chain	Residues	Atoms	ZeroOcc	AltConf
2	A	1	Total C N O P<br>22 10 3 8 1	0	0

- Molecule 3 is (2R)-2,3-dihydroxypropyl (9Z)-octadec-9-enoate (three-letter code: OLC) (formula: $C_{21}H_{40}O_4$).

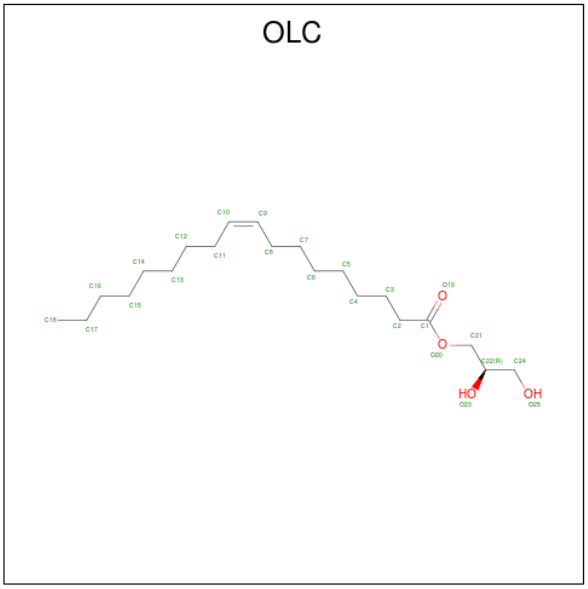

Mol	Chain	Residues	Atoms	ZeroOcc	AltConf
3	A	1	Total C O<br>15 11 4	0	0
3	A	1	Total C O<br>15 11 4	0	0
3	A	1	Total C O<br>8 6 2	0	0
3	A	1	Total C O<br>12 8 4	0	0
3	A	1	Total C O<br>12 8 4	0	0
3	A	1	Total C O<br>9 5 4	0	0
3	A	1	Total C O<br>20 16 4	0	0
3	A	1	Total C O<br>14 10 4	0	0
3	A	1	Total C O<br>21 17 4	0	0
3	A	1	Total C O<br>14 10 4	0	0

Continued on next page...

CONFIDENTIAL VALIDATION REPORT

Continued from previous page...

Mol	Chain	Residues	Atoms	ZeroOcc	AltConf
3	A	1	Total C O<br>12 8 4	0	0
3	A	1	Total C O<br>9 5 4	0	0
3	A	1	Total C O<br>13 11 2	0	0

- Molecule 4 is NONAETHYLENE GLYCOL (three-letter code: 2PE) (formula: $C_{18}H_{38}O_{10}$).

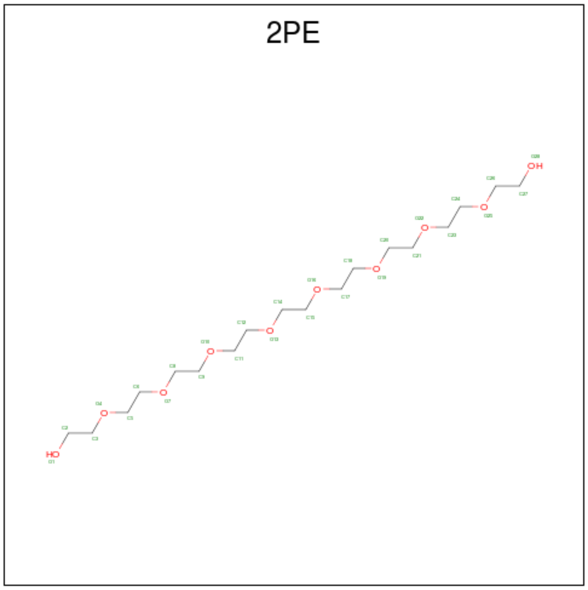

Mol	Chain	Residues	Atoms	ZeroOcc	AltConf
4	A	1	Total C O<br>10 6 4	0	0
4	A	1	Total C O<br>10 6 4	0	0

- Molecule 5 is water.

Mol	Chain	Residues	Atoms	ZeroOcc	AltConf
5	A	162	Total O<br>162 162	0	0

CONFIDENTIAL VALIDATION REPORT

3 Residue-property plots (i)

These plots are drawn for all protein, RNA and DNA chains in the entry. The first graphic for a chain summarises the proportions of the various outlier classes displayed in the second graphic. The second graphic shows the sequence view annotated by issues in geometry and electron density. Residues are color-coded according to the number of geometric quality criteria for which they contain at least one outlier: green = 0, yellow = 1, orange = 2 and red = 3 or more. A red dot above a residue indicates a poor fit to the electron density (RSRZ > 2). Stretches of 2 or more consecutive residues without any outlier are shown as a green connector. Residues present in the sample, but not in the model, are shown in grey.

- Molecule 1: CMP-sialic acid transporter

Chain A:
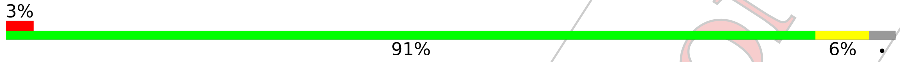

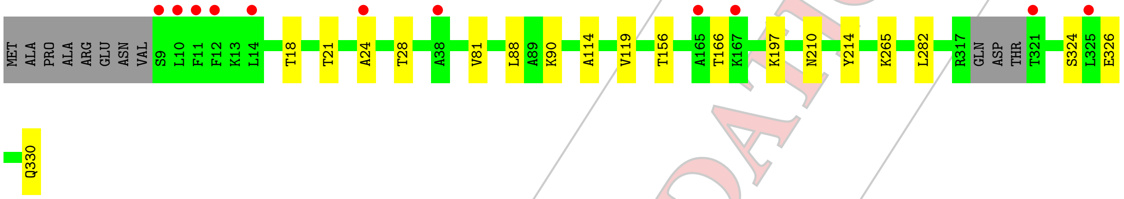

CONFIDENTIAL VALIDATION REPORT

4 Data and refinement statistics (i)

Property	Value	Source
Space group	C 1 2 1	Depositor
Cell constants<br>a, b, c, α, β, γ	144.26Å 49.40Å 49.83Å<br>90.00° 107.62° 90.00°	Depositor
Resolution (Å)	46.15 – 1.80<br>47.49 – 1.80	Depositor<br>EDS
% Data completeness<br>(in resolution range)	99.9 (46.15-1.80)<br>100.0 (47.49-1.80)	Depositor<br>EDS
R_{merge}	0.13	Depositor
R_{sym}	(Not available)	Depositor
$< I/\sigma(I) >$ [1]	1.00 (at 1.79Å)	Xtriage
Refinement program	PHENIX 1.14_3260	Depositor
R, R_{free}	0.180 , 0.198<br>0.181 , 0.200	Depositor<br>DCC
R_{free} test set	1582 reflections (5.07%)	wwPDB-VP
Wilson B-factor (Å^2)	37.1	Xtriage
Anisotropy	0.165	Xtriage
Bulk solvent k_{sol}(e/Å^3), B_{sol}(Å^2)	0.34 , 74.9	EDS
L-test for twinning[2]	$< \|L\| > = 0.50$, $< L^2 > = 0.34$	Xtriage
Estimated twinning fraction	0.022 for -h+k-l,-l,-k<br>0.000 for -h-k-l,l,k<br>0.023 for -h-2*l,-k,l	Xtriage
F_o,F_c correlation	0.96	EDS
Total number of atoms	2841	wwPDB-VP
Average B, all atoms (Å^2)	42.0	wwPDB-VP

Xtriage's analysis on translational NCS is as follows: *The largest off-origin peak in the Patterson function is 8.60% of the height of the origin peak. No significant pseudotranslation is detected.*

[1] Intensities estimated from amplitudes.

[2] Theoretical values of $< |L| >$, $< L^2 >$ for acentric reflections are 0.5, 0.333 respectively for untwinned datasets, and 0.375, 0.2 for perfectly twinned datasets.

CONFIDENTIAL VALIDATION REPORT

5 Model quality (i)

5.1 Standard geometry (i)

Bond lengths and bond angles in the following residue types are not validated in this section: OLC, 2PE, 5MC

The Z score for a bond length (or angle) is the number of standard deviations the observed value is removed from the expected value. A bond length (or angle) with $|Z| > 5$ is considered an outlier worth inspection. RMSZ is the root-mean-square of all Z scores of the bond lengths (or angles).

Mol	Chain	Bond lengths		Bond angles	
		RMSZ	$\#|Z| > 5$	RMSZ	$\#|Z| > 5$
1	A	0.36	0/2508	0.47	0/3419

There are no bond length outliers.

There are no bond angle outliers.

There are no chirality outliers.

There are no planarity outliers.

5.2 Too-close contacts (i)

In the following table, the Non-H and H(model) columns list the number of non-hydrogen atoms and hydrogen atoms in the chain respectively. The H(added) column lists the number of hydrogen atoms added and optimized by MolProbity. The Clashes column lists the number of clashes within the asymmetric unit, whereas Symm-Clashes lists symmetry related clashes.

Mol	Chain	Non-H	H(model)	H(added)	Clashes	Symm-Clashes
1	A	2463	0	2529	13	0
2	A	22	0	14	0	0
3	A	174	0	205	3	0
4	A	20	0	26	2	0
5	A	162	0	0	2	2
All	All	2841	0	2774	13	2

The all-atom clashscore is defined as the number of clashes found per 1000 atoms (including hydrogen atoms). The all-atom clashscore for this structure is 2.

All (13) close contacts within the same asymmetric unit are listed below, sorted by their clash magnitude.

CONFIDENTIAL VALIDATION REPORT

Atom-1	Atom-2	Interatomic distance (Å)	Clash overlap (Å)
1:A:197:LYS:HD3	3:A:412:OLC:H24	1.86	0.58
1:A:265:LYS:HZ2	4:A:415:2PE:H91	1.70	0.56
1:A:119:VAL:HG13	1:A:156:THR:HG23	1.90	0.53
1:A:326:GLU:O	1:A:330:GLN:HG2	2.09	0.52
1:A:18:THR:HA	1:A:21:THR:HG22	1.92	0.51
1:A:24:ALA:HB2	5:A:592:HOH:O	2.13	0.47
1:A:81:VAL:HG11	3:A:414:OLC:H4A	1.99	0.43
1:A:88:LEU:HB3	3:A:408:OLC:H6A	2.00	0.43
1:A:28:THR:HG22	1:A:114:ALA:HB1	2.02	0.42
1:A:90:LYS:NZ	5:A:511:HOH:O	2.52	0.40
1:A:210:ASN:HB3	1:A:214:TYR:CE2	2.57	0.40
1:A:265:LYS:NZ	4:A:415:2PE:H91	2.35	0.40
1:A:282:LEU:HA	1:A:282:LEU:HD23	1.95	0.40

All (2) symmetry-related close contacts are listed below. The label for Atom-2 includes the symmetry operator and encoded unit-cell translations to be applied.

Atom-1	Atom-2	Interatomic distance (Å)	Clash overlap (Å)
5:A:641:HOH:O	5:A:655:HOH:O[1_565]	2.14	0.06
5:A:640:HOH:O	5:A:643:HOH:O[4_555]	2.18	0.02

5.3 Torsion angles (i)

5.3.1 Protein backbone (i)

In the following table, the Percentiles column shows the percent Ramachandran outliers of the chain as a percentile score with respect to all X-ray entries followed by that with respect to entries of similar resolution.

The Analysed column shows the number of residues for which the backbone conformation was analysed, and the total number of residues.

Mol	Chain	Analysed	Favoured	Allowed	Outliers	Percentiles	
1	A	325/330 (98%)	321 (99%)	4 (1%)	0	100	100

There are no Ramachandran outliers to report.

5.3.2 Protein sidechains (i)

In the following table, the Percentiles column shows the percent sidechain outliers of the chain as a percentile score with respect to all X-ray entries followed by that with respect to entries of similar

CONFIDENTIAL VALIDATION REPORT

resolution.

The Analysed column shows the number of residues for which the sidechain conformation was analysed, and the total number of residues.

Mol	Chain	Analysed	Rotameric	Outliers	Percentiles	
1	A	265/275 (96%)	263 (99%)	2 (1%)	81	78

All (2) residues with a non-rotameric sidechain are listed below:

Mol	Chain	Res	Type
1	A	166	THR
1	A	324	SER

Some sidechains can be flipped to improve hydrogen bonding and reduce clashes. There are no such sidechains identified.

5.3.3 RNA (i)

There are no RNA molecules in this entry.

5.4 Non-standard residues in protein, DNA, RNA chains (i)

There are no non-standard protein/DNA/RNA residues in this entry.

5.5 Carbohydrates (i)

There are no carbohydrates in this entry.

5.6 Ligand geometry (i)

16 ligands are modelled in this entry.

In the following table, the Counts columns list the number of bonds (or angles) for which Mogul statistics could be retrieved, the number of bonds (or angles) that are observed in the model and the number of bonds (or angles) that are defined in the Chemical Component Dictionary. The Link column lists molecule types, if any, to which the group is linked. The Z score for a bond length (or angle) is the number of standard deviations the observed value is removed from the expected value. A bond length (or angle) with $|Z| > 2$ is considered an outlier worth inspection. RMSZ is the root-mean-square of all Z scores of the bond lengths (or angles).

CONFIDENTIAL VALIDATION REPORT

Mol	Type	Chain	Res	Link	Bond lengths			Bond angles		
					Counts	RMSZ	#|Z| > 2	Counts	RMSZ	#|Z| > 2
4	2PE	A	415	-	9,9,27	0.47	0	8,8,26	0.80	0
3	OLC	A	413	-	8,8,24	0.38	0	9,9,25	0.92	0
3	OLC	A	414	-	12,12,24	0.62	0	11,11,25	1.02	0
3	OLC	A	407	-	8,8,24	0.36	0	9,9,25	0.85	0
3	OLC	A	403	-	14,14,24	0.47	0	15,15,25	0.93	1 (6%)
2	5MC	A	401	-	19,23,23	0.95	1 (5%)	26,35,35	1.35	3 (11%)
4	2PE	A	416	-	9,9,27	0.57	0	8,8,26	0.64	0
3	OLC	A	409	-	13,13,24	0.36	0	14,14,25	1.19	1 (7%)
3	OLC	A	406	-	11,11,24	0.45	0	12,12,25	0.79	0
3	OLC	A	411	-	13,13,24	0.42	0	14,14,25	1.10	1 (7%)
3	OLC	A	405	-	11,11,24	0.36	0	12,12,25	1.10	1 (8%)
3	OLC	A	402	-	14,14,24	0.42	0	15,15,25	0.93	1 (6%)
3	OLC	A	408	-	19,19,24	0.36	0	20,20,25	1.09	1 (5%)
3	OLC	A	412	-	11,11,24	0.40	0	12,12,25	1.01	1 (8%)
3	OLC	A	410	-	20,20,24	0.36	0	21,21,25	0.95	1 (4%)
3	OLC	A	404	-	7,7,24	0.77	0	6,6,25	1.25	1 (16%)

In the following table, the Chirals column lists the number of chiral outliers, the number of chiral centers analysed, the number of these observed in the model and the number defined in the Chemical Component Dictionary. Similar counts are reported in the Torsion and Rings columns. '-' means no outliers of that kind were identified.

Mol	Type	Chain	Res	Link	Chirals	Torsions	Rings
4	2PE	A	415	-	-	2/7/7/25	-
3	OLC	A	413	-	-	2/7/7/24	-
3	OLC	A	414	-	-	5/10/10/24	-
3	OLC	A	407	-	-	6/7/7/24	-
3	OLC	A	403	-	-	3/14/14/24	-
2	5MC	A	401	-	-	1/8/26/26	0/2/2/2
4	2PE	A	416	-	-	6/7/7/25	-
3	OLC	A	409	-	-	2/13/13/24	-
3	OLC	A	406	-	-	3/11/11/24	-
3	OLC	A	411	-	-	2/13/13/24	-
3	OLC	A	405	-	-	2/11/11/24	-
3	OLC	A	402	-	-	5/14/14/24	-
3	OLC	A	408	-	-	7/19/19/24	-
3	OLC	A	412	-	-	7/11/11/24	-

Continued on next page...

CONFIDENTIAL VALIDATION REPORT

Continued from previous page...

Mol	Type	Chain	Res	Link	Chirals	Torsions	Rings
3	OLC	A	410	-	-	9/20/20/24	-
3	OLC	A	404	-	-	1/5/5/24	-

All (1) bond length outliers are listed below:

Mol	Chain	Res	Type	Atoms	Z	Observed(Å)	Ideal(Å)
2	A	401	5MC	C2-N3	-2.20	1.33	1.38

All (12) bond angle outliers are listed below:

Mol	Chain	Res	Type	Atoms	Z	Observed(^{o})	Ideal(^{o})
2	A	401	5MC	C2-N3-C4	4.11	120.98	116.02
3	A	408	OLC	O20-C1-C2	3.13	121.73	111.91
2	A	401	5MC	C5-C6-N1	-2.99	118.97	122.19
3	A	409	OLC	O20-C1-C2	2.98	121.25	111.91
3	A	410	OLC	O20-C1-C2	2.84	120.81	111.91
3	A	411	OLC	O20-C1-C2	2.78	120.62	111.91
3	A	405	OLC	O20-C1-C2	2.74	120.50	111.91
3	A	404	OLC	C21-C22-C24	-2.63	108.21	113.95
3	A	402	OLC	O20-C1-C2	2.53	119.86	111.91
3	A	412	OLC	O20-C1-C2	2.50	119.76	111.91
3	A	403	OLC	O20-C1-C2	2.39	119.40	111.91
2	A	401	5MC	OP2-P-OP1	2.12	118.97	110.68

There are no chirality outliers.

All (63) torsion outliers are listed below:

Mol	Chain	Res	Type	Atoms
3	A	407	OLC	O20-C21-C22-C24
3	A	409	OLC	C21-C22-C24-O25
3	A	406	OLC	O20-C21-C22-C24
3	A	411	OLC	C21-C22-C24-O25
3	A	402	OLC	O20-C21-C22-O23
3	A	412	OLC	O20-C21-C22-C24
3	A	408	OLC	C2-C1-O20-C21
3	A	408	OLC	O19-C1-O20-C21
3	A	407	OLC	O20-C21-C22-O23
3	A	406	OLC	O20-C21-C22-O23
4	A	416	2PE	O4-C5-C6-O7
3	A	407	OLC	C2-C1-O20-C21
3	A	412	OLC	C2-C1-O20-C21

Continued on next page...

CONFIDENTIAL VALIDATION REPORT

Continued from previous page...

Mol	Chain	Res	Type	Atoms
3	A	412	OLC	O20-C21-C22-O23
3	A	410	OLC	O20-C21-C22-O23
3	A	414	OLC	O20-C21-C22-C24
3	A	412	OLC	O19-C1-O20-C21
3	A	405	OLC	O20-C21-C22-O23
3	A	412	OLC	C1-C2-C3-C4
3	A	407	OLC	O19-C1-O20-C21
3	A	410	OLC	O20-C21-C22-C24
3	A	403	OLC	C4-C5-C6-C7
3	A	410	OLC	C2-C3-C4-C5
3	A	413	OLC	C21-C22-C24-O25
3	A	407	OLC	C21-C22-C24-O25
3	A	408	OLC	C21-C22-C24-O25
3	A	412	OLC	C21-C22-C24-O25
3	A	410	OLC	C21-C22-C24-O25
3	A	410	OLC	C4-C5-C6-C7
3	A	408	OLC	C4-C5-C6-C7
3	A	407	OLC	O23-C22-C24-O25
3	A	411	OLC	O23-C22-C24-O25
3	A	412	OLC	O23-C22-C24-O25
4	A	415	2PE	O4-C5-C6-O7
3	A	402	OLC	O20-C21-C22-C24
3	A	414	OLC	O20-C1-C2-C3
3	A	402	OLC	C3-C4-C5-C6
3	A	413	OLC	O23-C22-C24-O25
3	A	409	OLC	O23-C22-C24-O25
3	A	408	OLC	O23-C22-C24-O25
3	A	410	OLC	O23-C22-C24-O25
3	A	414	OLC	C5-C6-C7-C8
3	A	402	OLC	C5-C6-C7-C8
3	A	403	OLC	C2-C1-O20-C21
3	A	403	OLC	O19-C1-O20-C21
3	A	408	OLC	C6-C7-C8-C9
3	A	404	OLC	C22-C21-O20-C1
2	A	401	5MC	O4'-C4'-C5'-O5'
3	A	410	OLC	O19-C1-O20-C21
3	A	410	OLC	C2-C1-O20-C21
3	A	414	OLC	C4-C5-C6-C7
4	A	416	2PE	C6-C5-O4-C3
4	A	416	2PE	C9-C8-O7-C6
4	A	415	2PE	C2-C3-O4-C5
3	A	408	OLC	C7-C8-C9-C10

Continued on next page...

CONFIDENTIAL VALIDATION REPORT

Continued from previous page...

Mol	Chain	Res	Type	Atoms
3	A	405	OLC	O20-C21-C22-C24
4	A	416	2PE	O1-C2-C3-O4
3	A	402	OLC	C4-C5-C6-C7
3	A	410	OLC	C7-C8-C9-C10
4	A	416	2PE	C5-C6-O7-C8
3	A	406	OLC	O23-C22-C24-O25
3	A	414	OLC	C2-C1-O20-C21
4	A	416	2PE	C2-C3-O4-C5

There are no ring outliers.

4 monomers are involved in 5 short contacts:

Mol	Chain	Res	Type	Clashes	Symm-Clashes
4	A	415	2PE	2	0
3	A	414	OLC	1	0
3	A	408	OLC	1	0
3	A	412	OLC	1	0

The following is a two-dimensional graphical depiction of Mogul quality analysis of bond lengths, bond angles, torsion angles, and ring geometry for all instances of the Ligand of Interest. In addition, ligands with molecular weight > 250 and outliers as shown on the validation Tables will also be included. For torsion angles, if less then 5% of the Mogul distribution of torsion angles is within 10 degrees of the torsion angle in question, then that torsion angle is considered an outlier. Any bond that is central to one or more torsion angles identified as an outlier by Mogul will be highlighted in the graph. For rings, the root-mean-square deviation (RMSD) between the ring in question and similar rings identified by Mogul is calculated over all ring torsion angles. If the average RMSD is greater than 60 degrees and the minimal RMSD between the ring in question and any Mogul-identified rings is also greater than 60 degrees, then that ring is considered an outlier. The outliers are highlighted in purple. The color gray indicates Mogul did not find sufficient equivalents in the CSD to analyse the geometry.

Ligand 2PE A 415

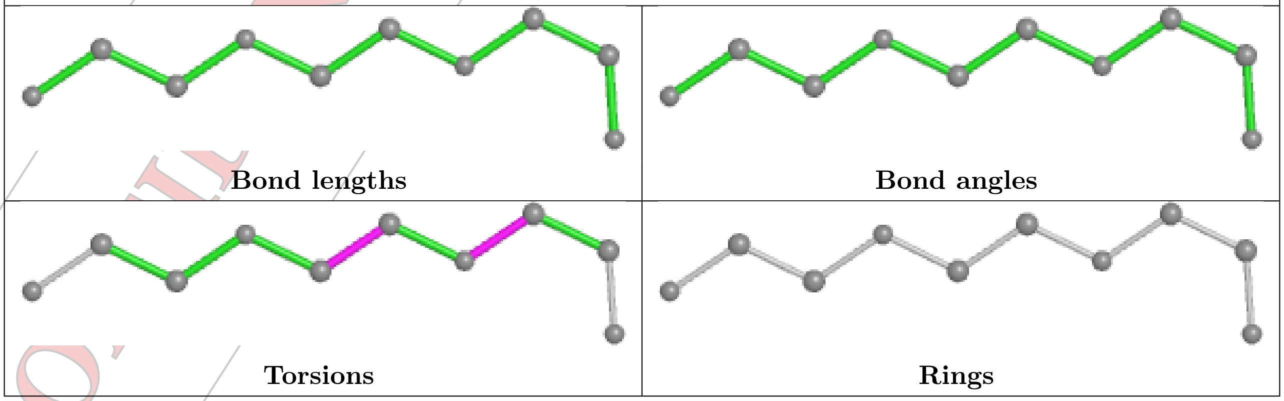

CONFIDENTIAL VALIDATION REPORT

Ligand OLC A 413

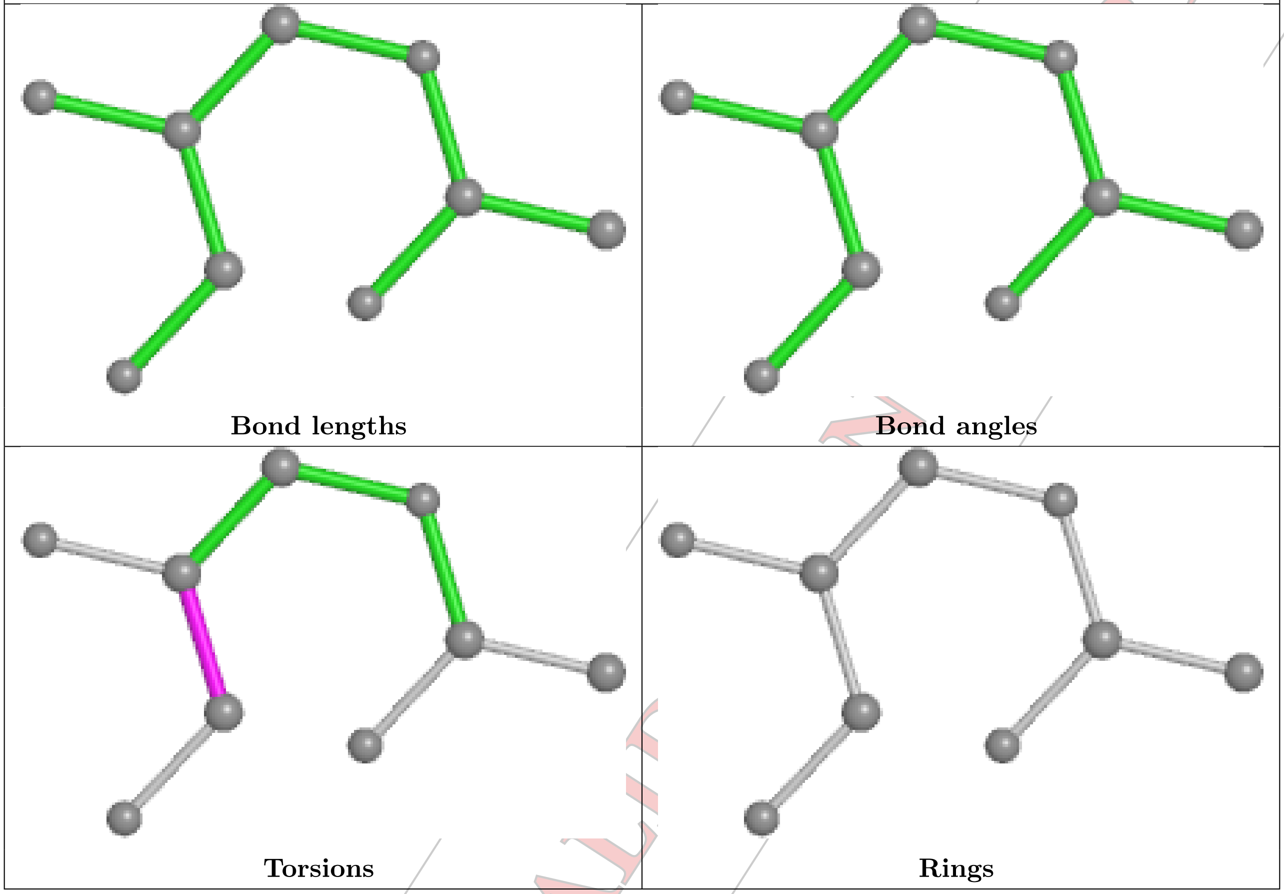

Ligand OLC A 414

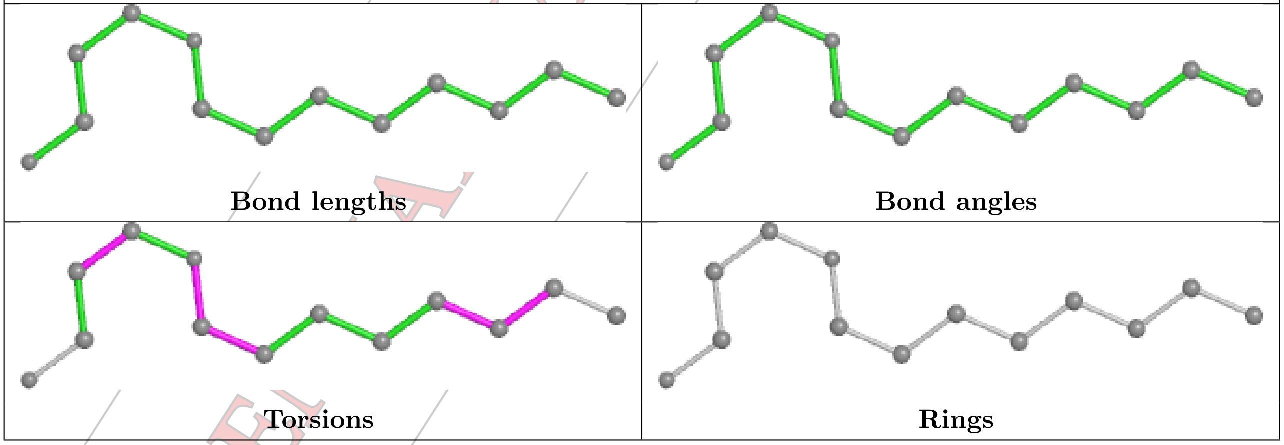

Ligand OLC A 407

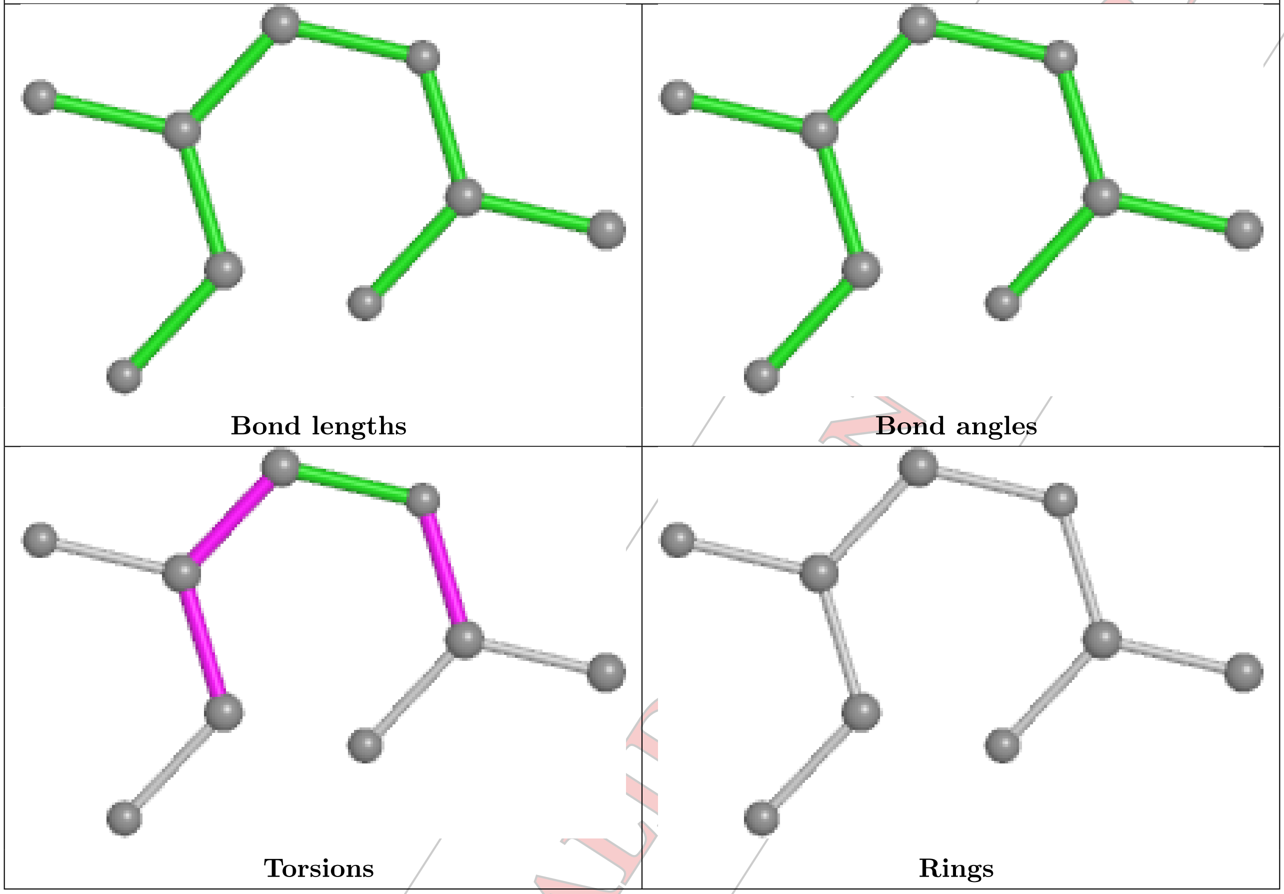

Ligand OLC A 403

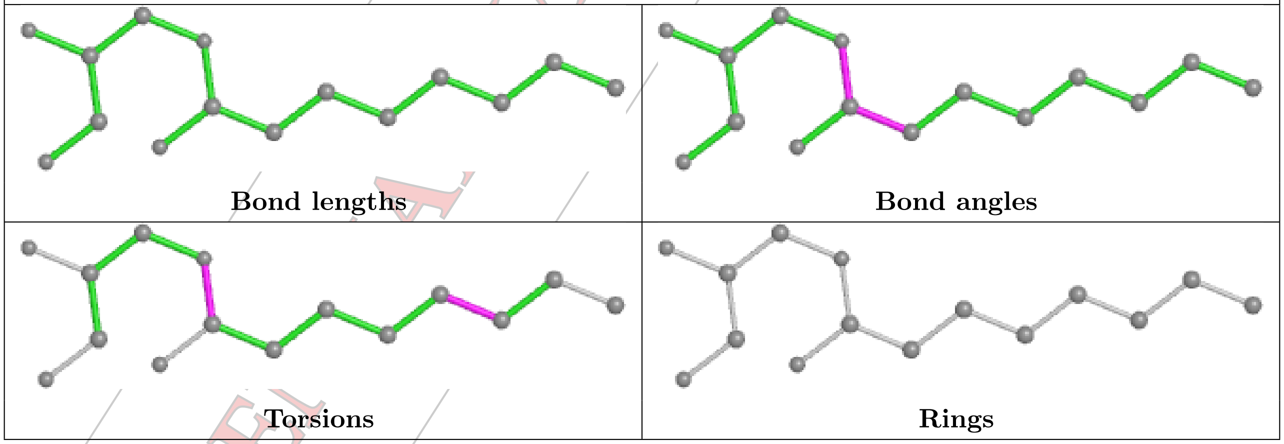

Ligand 5MC A 401

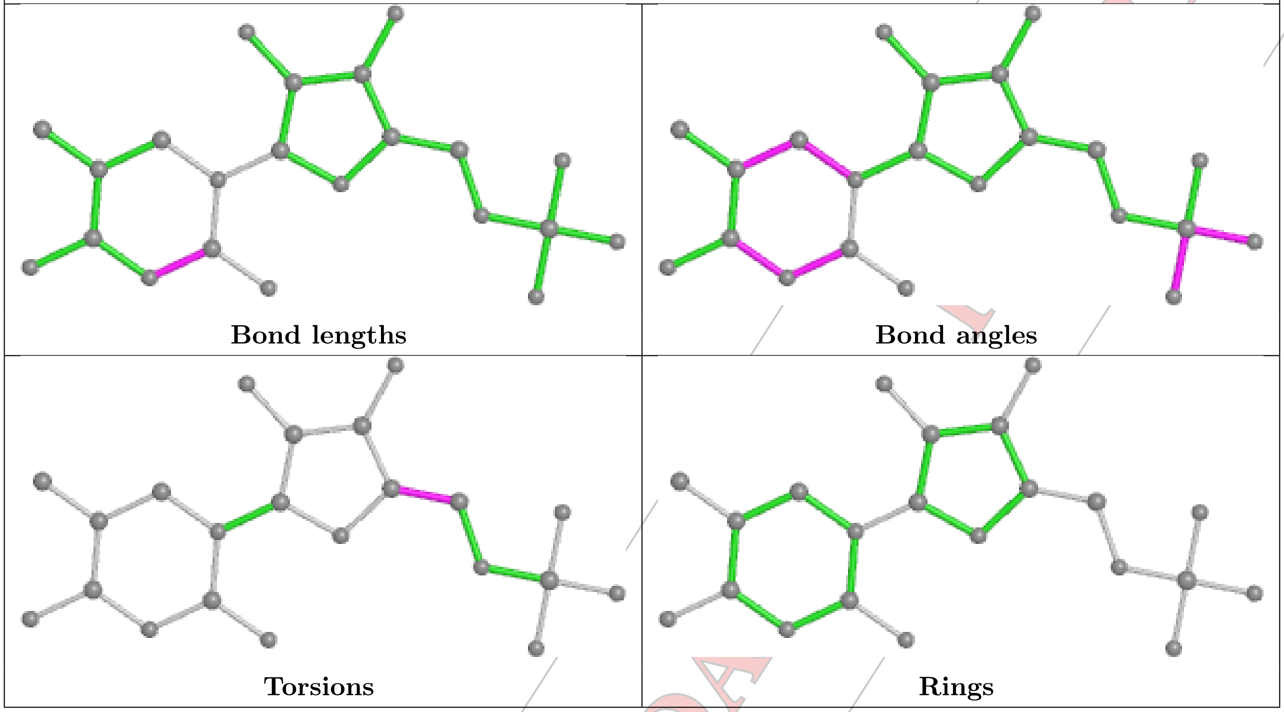

Ligand 2PE A 416

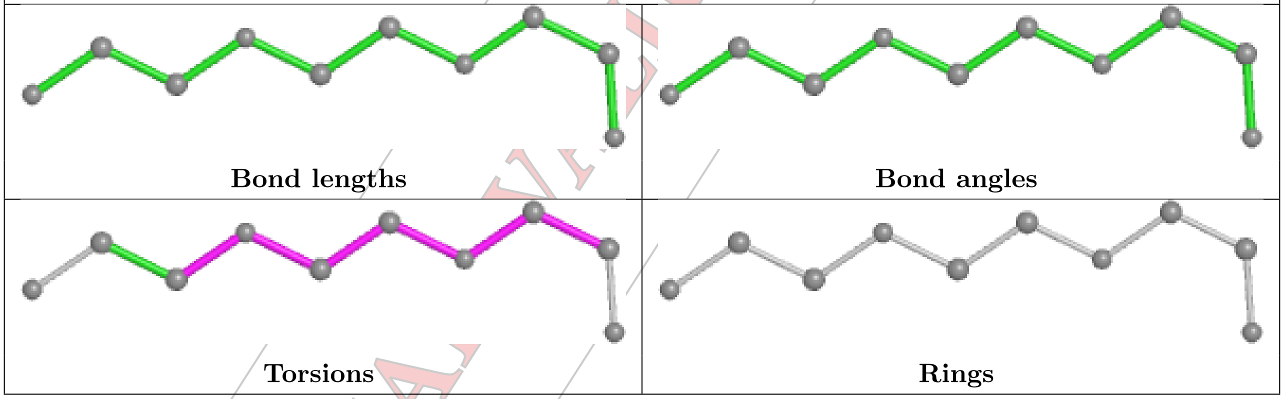

Ligand OLC A 409

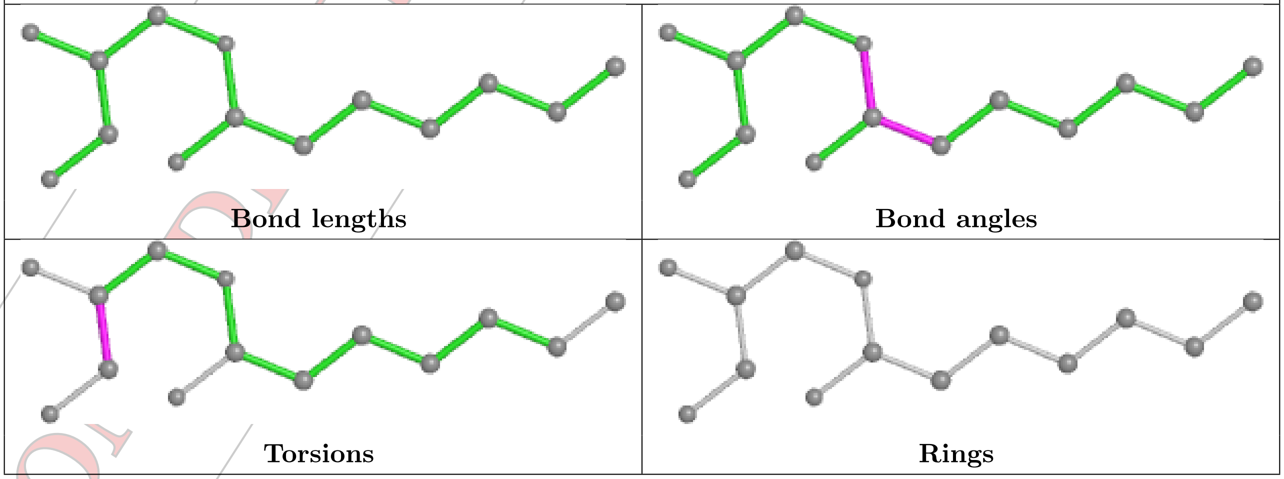

Ligand OLC A 406

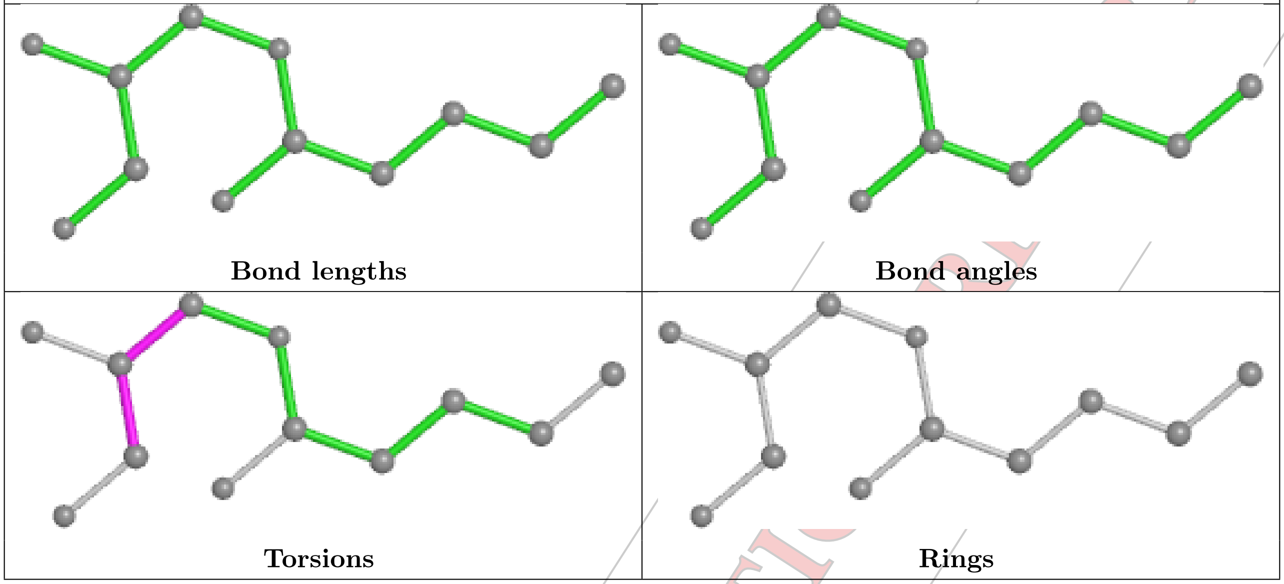

Ligand OLC A 411

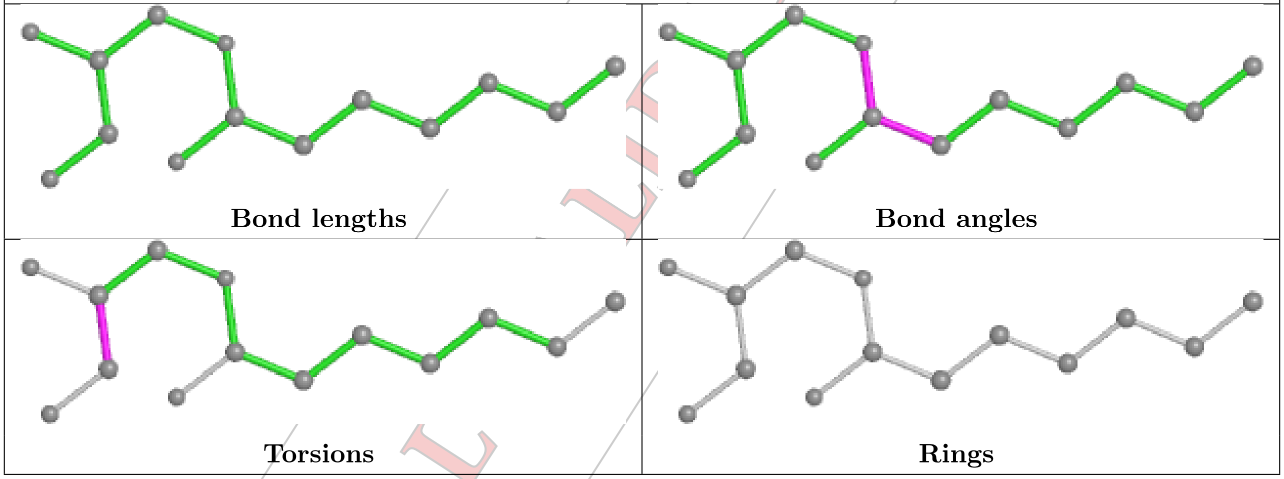

Ligand OLC A 405

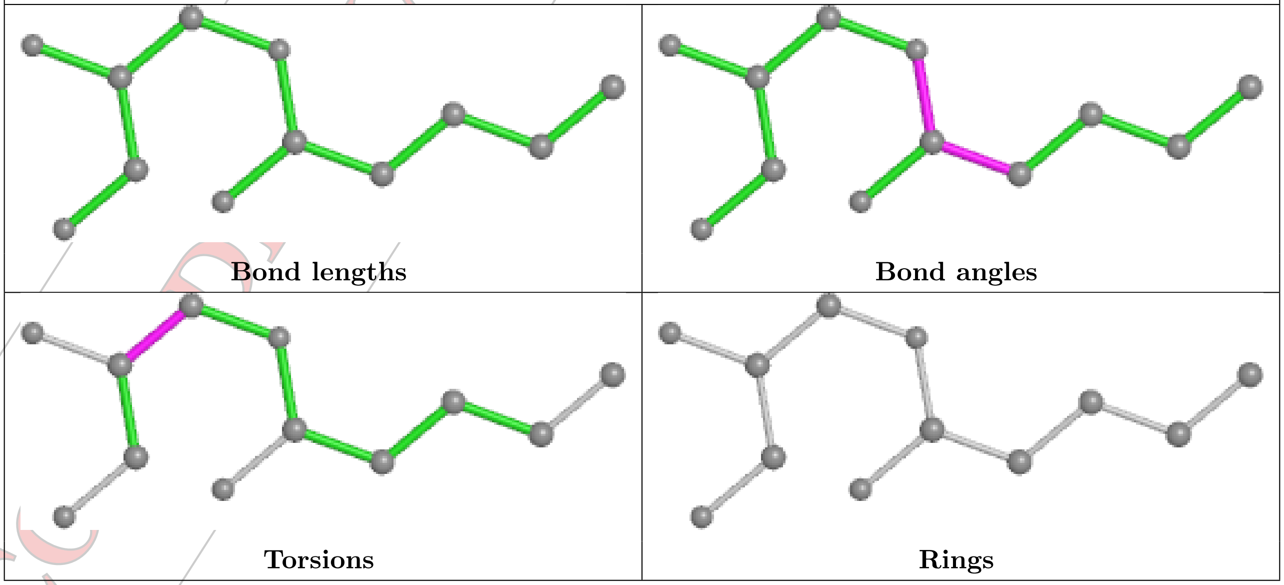

Ligand OLC A 402

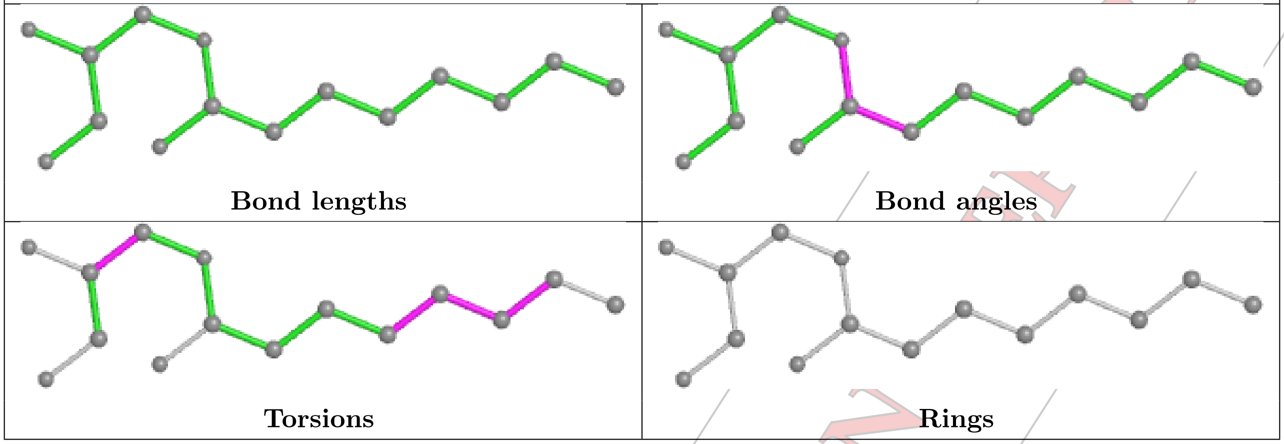

Ligand OLC A 408

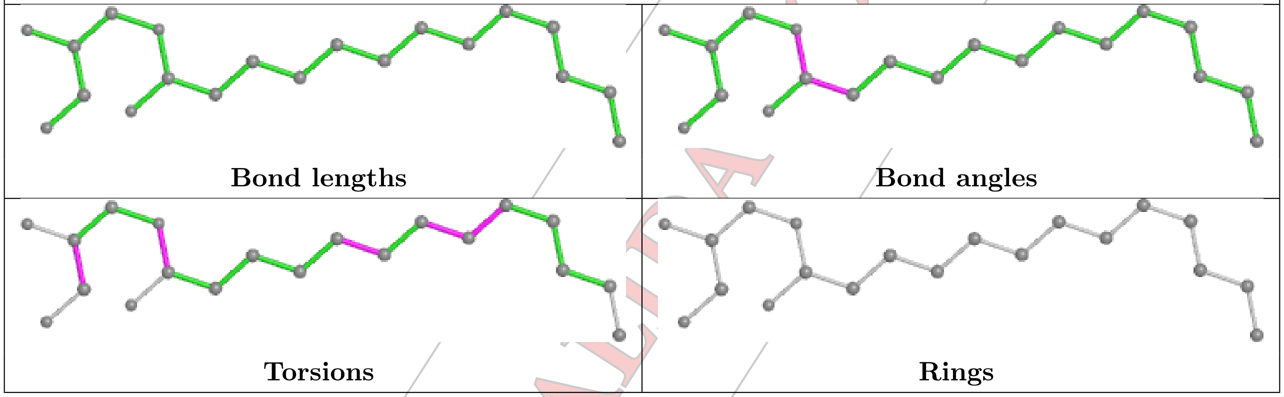

Ligand OLC A 412

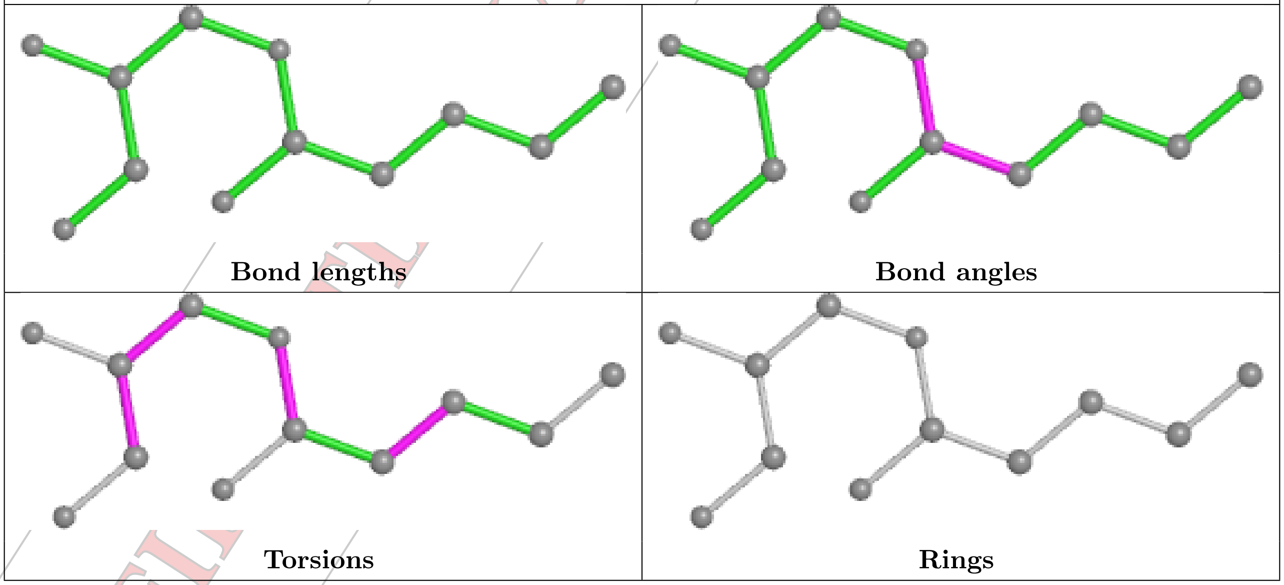

Ligand OLC A 410

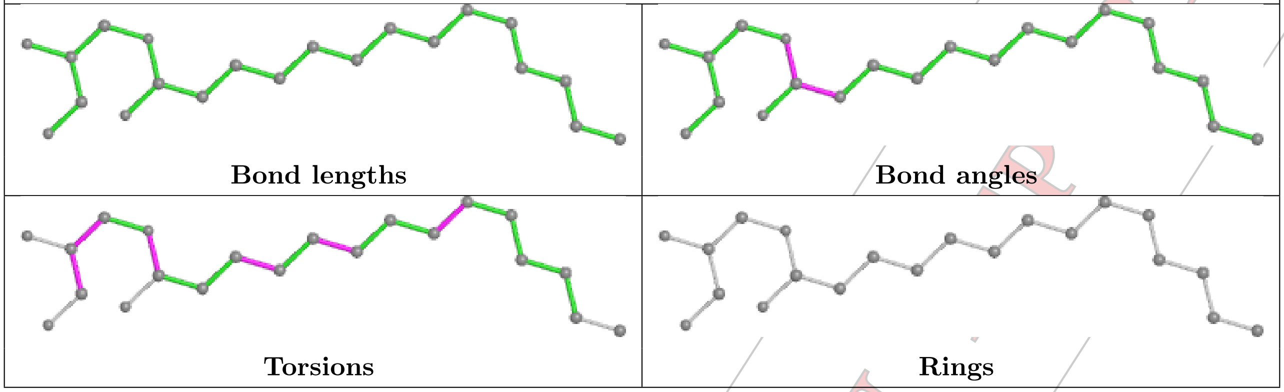

Ligand OLC A 404

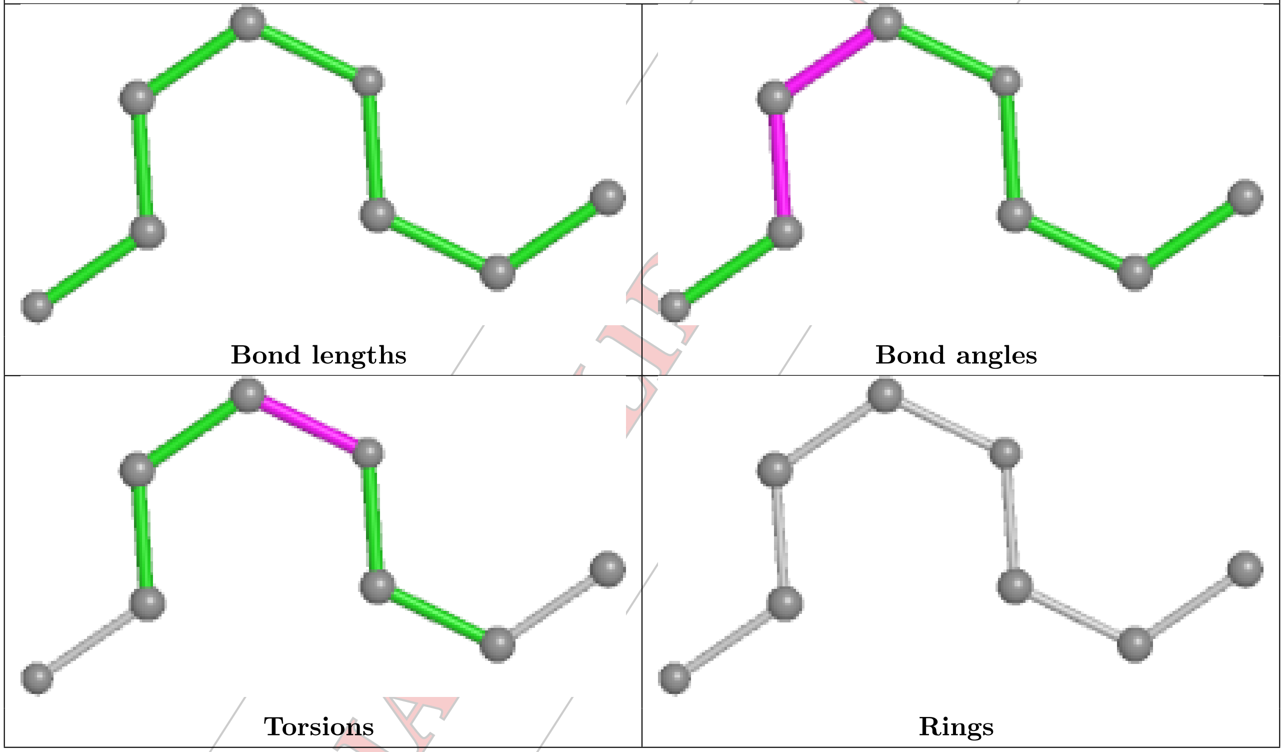

5.7 Other polymers (i)

There are no such residues in this entry.

5.8 Polymer linkage issues (i)

There are no chain breaks in this entry.

CONFIDENTIAL REPORT

6 Fit of model and data (i)

6.1 Protein, DNA and RNA chains (i)

In the following table, the column labelled '#RSRZ> 2' contains the number (and percentage) of RSRZ outliers, followed by percent RSRZ outliers for the chain as percentile scores relative to all X-ray entries and entries of similar resolution. The OWAB column contains the minimum, median, 95th percentile and maximum values of the occupancy-weighted average B-factor per residue. The column labelled 'Q< 0.9' lists the number of (and percentage) of residues with an average occupancy less than 0.9.

Mol	Chain	Analysed	<RSRZ>	#RSRZ>2			OWAB(Å^2)	Q<0.9
1	A	319/330 (96%)	-0.35	11 (3%)	45	39	29, 37, 66, 81	0

All (11) RSRZ outliers are listed below:

Mol	Chain	Res	Type	RSRZ
1	A	10	LEU	5.3
1	A	165	ALA	4.8
1	A	12	PHE	4.5
1	A	11	PHE	4.4
1	A	14	LEU	4.1
1	A	321	THR	3.1
1	A	38	ALA	2.9
1	A	325	LEU	2.5
1	A	24	ALA	2.3
1	A	9	SER	2.3
1	A	167	LYS	2.1

6.2 Non-standard residues in protein, DNA, RNA chains (i)

There are no non-standard protein/DNA/RNA residues in this entry.

6.3 Carbohydrates (i)

There are no carbohydrates in this entry.

6.4 Ligands (i)

In the following table, the Atoms column lists the number of modelled atoms in the group and the number defined in the chemical component dictionary. The B-factors column lists the minimum,

CONFIDENTIAL VALIDATION REPORT

median, 95th percentile and maximum values of B factors of atoms in the group. The column labelled 'Q< 0.9' lists the number of atoms with occupancy less than 0.9.

Mol	Type	Chain	Res	Atoms	RSCC	RSR	B-factors(Å^2)	Q<0.9
3	OLC	A	412	12/25	0.58	0.28	61,70,93,95	0
3	OLC	A	406	12/25	0.59	0.30	54,71,81,81	0
3	OLC	A	410	21/25	0.65	0.18	56,66,88,93	0
3	OLC	A	411	14/25	0.70	0.21	50,68,80,85	0
3	OLC	A	414	13/25	0.74	0.14	45,57,67,68	0
4	2PE	A	416	10/28	0.78	0.15	52,64,67,74	0
3	OLC	A	403	15/25	0.79	0.13	45,57,78,83	0
3	OLC	A	405	12/25	0.81	0.29	56,63,75,76	0
3	OLC	A	409	14/25	0.83	0.17	49,61,72,83	0
3	OLC	A	408	20/25	0.86	0.14	44,58,74,79	0
4	2PE	A	415	10/28	0.89	0.10	43,50,62,62	0
3	OLC	A	407	9/25	0.89	0.22	60,76,82,83	0
3	OLC	A	402	15/25	0.89	0.10	43,51,67,70	0
3	OLC	A	413	9/25	0.91	0.11	62,65,74,77	0
3	OLC	A	404	8/25	0.91	0.10	55,60,67,68	0
2	5MC	A	401	22/22	0.98	0.07	27,31,36,39	0

The following is a graphical depiction of the model fit to experimental electron density of all instances of the Ligand of Interest. In addition, ligands with molecular weight > 250 and outliers as shown on the geometry validation Tables will also be included. Each fit is shown from different orientation to approximate a three-dimensional view.

CONFIDENTIAL VALIDATION REPORT

Electron density around OLC A 412:

$2mF_o$-DF_c (at 0.7 rmsd) in gray
mF_o-DF_c (at 3 rmsd) in purple (negative)
and green (positive)

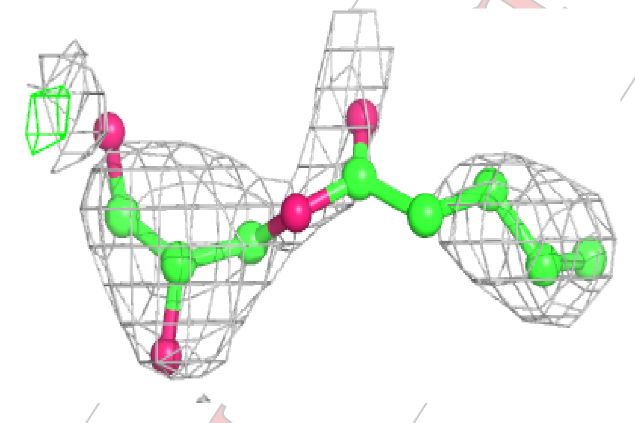

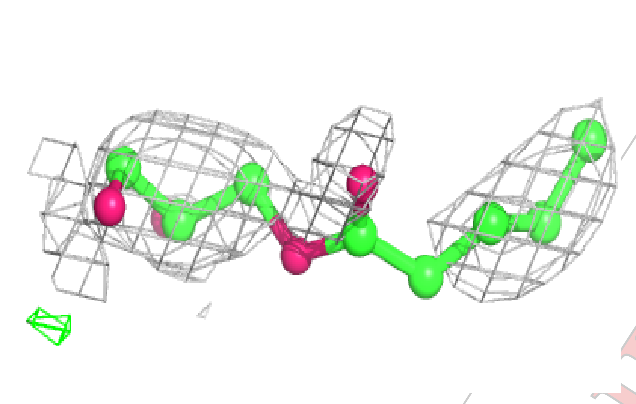

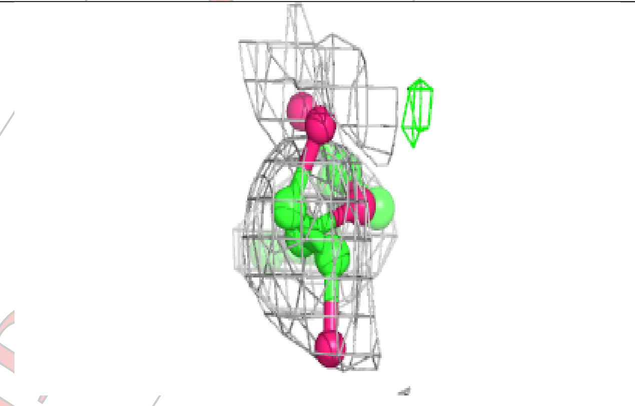

Electron density around OLC A 406:

$2mF_o$-DF_c (at 0.7 rmsd) in gray
mF_o-DF_c (at 3 rmsd) in purple (negative)
and green (positive)

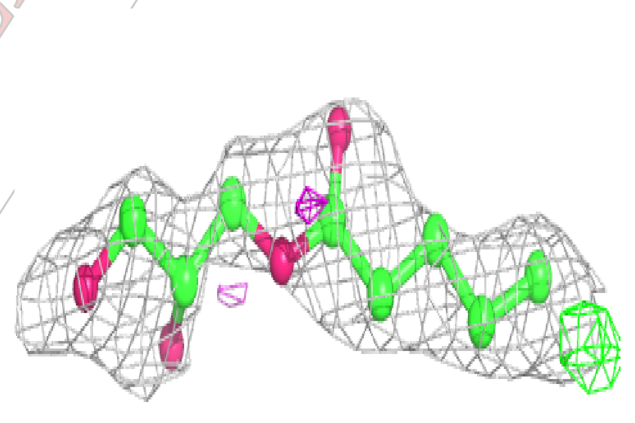

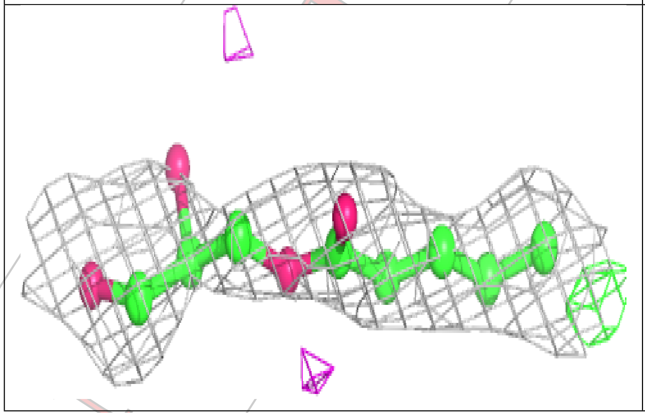

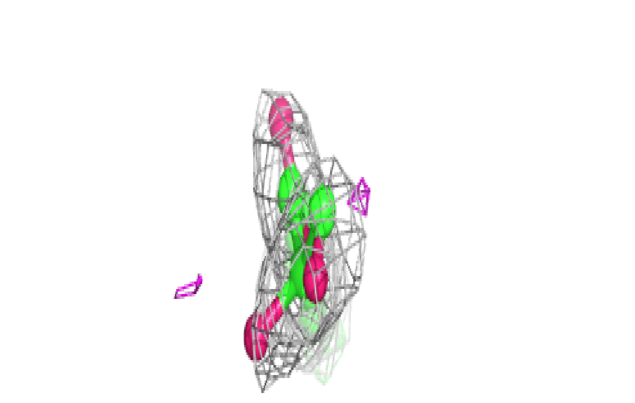

Electron density around OLC A 410:

$2mF_o$-DF_c (at 0.7 rmsd) in gray
mF_o-DF_c (at 3 rmsd) in purple (negative)
and green (positive)

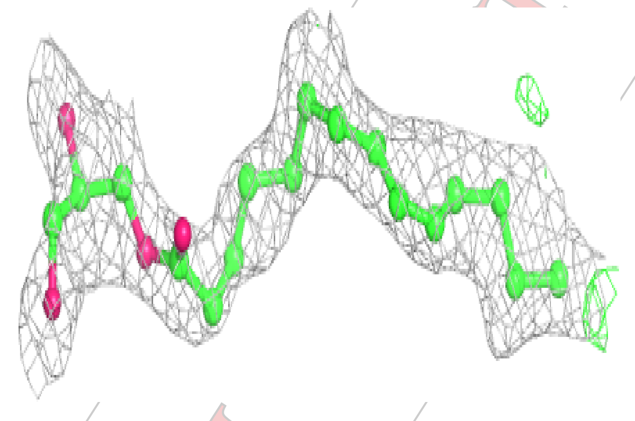

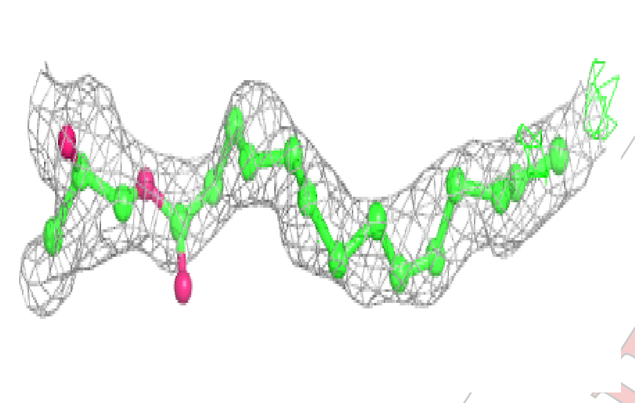

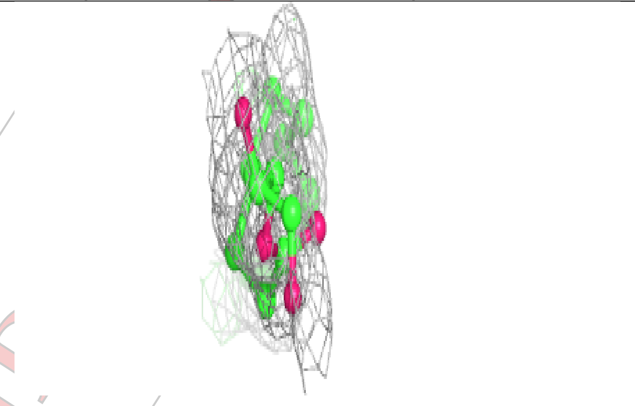

Electron density around OLC A 411:

$2mF_o$-DF_c (at 0.7 rmsd) in gray
mF_o-DF_c (at 3 rmsd) in purple (negative)
and green (positive)

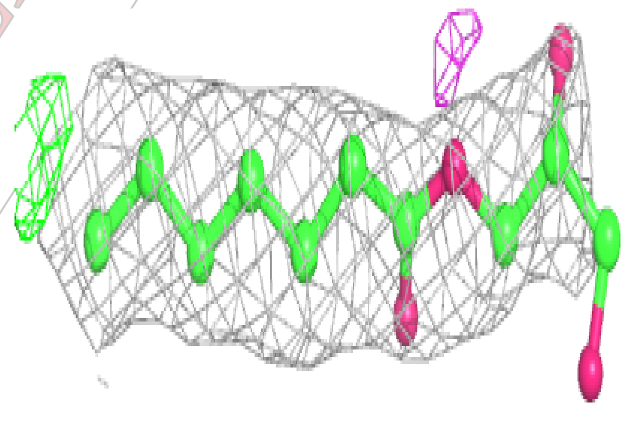

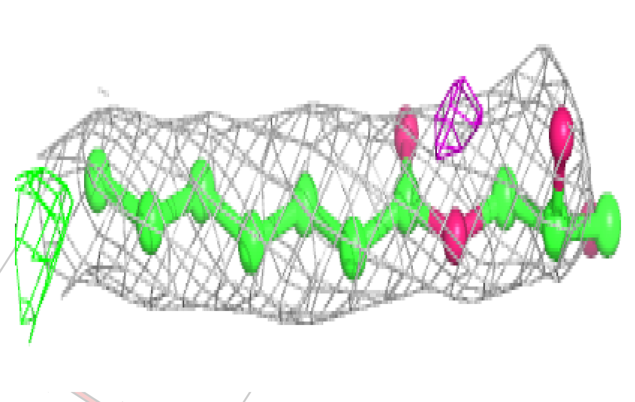

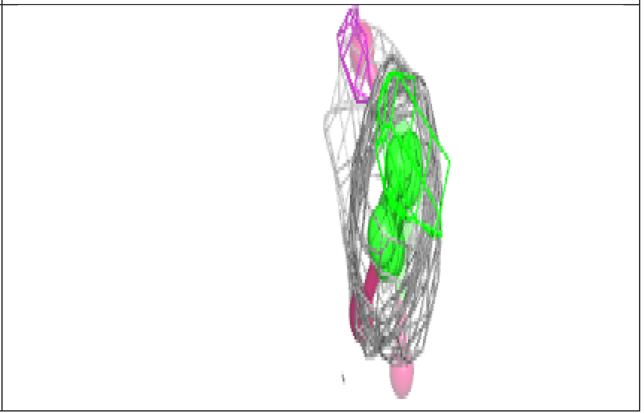

Electron density around OLC A 414:

2mF$_o$-DF$_c$ (at 0.7 rmsd) in gray
mF$_o$-DF$_c$ (at 3 rmsd) in purple (negative)
and green (positive)

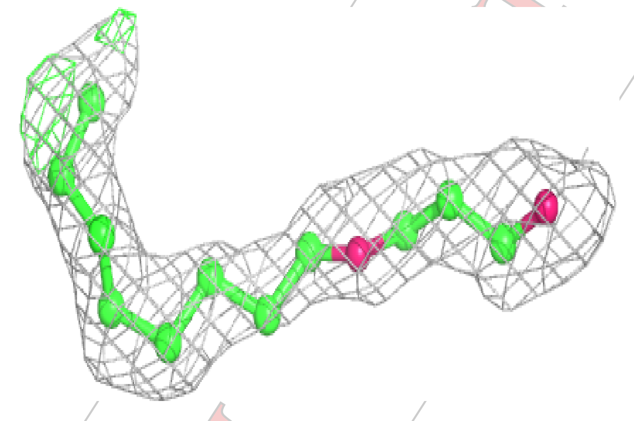

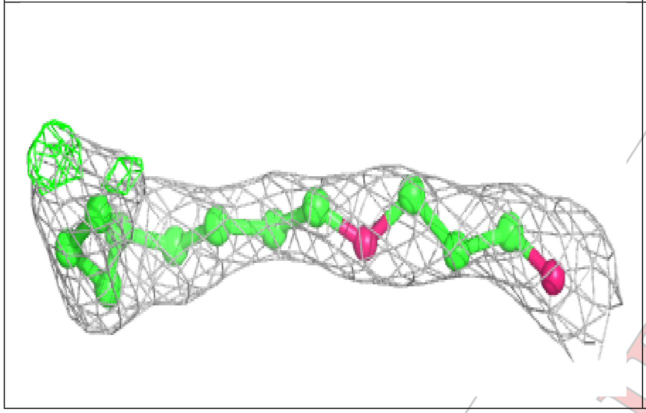

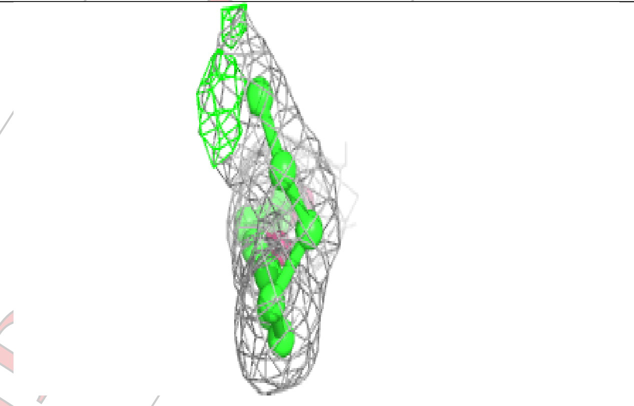

Electron density around 2PE A 416:

2mF$_o$-DF$_c$ (at 0.7 rmsd) in gray
mF$_o$-DF$_c$ (at 3 rmsd) in purple (negative)
and green (positive)

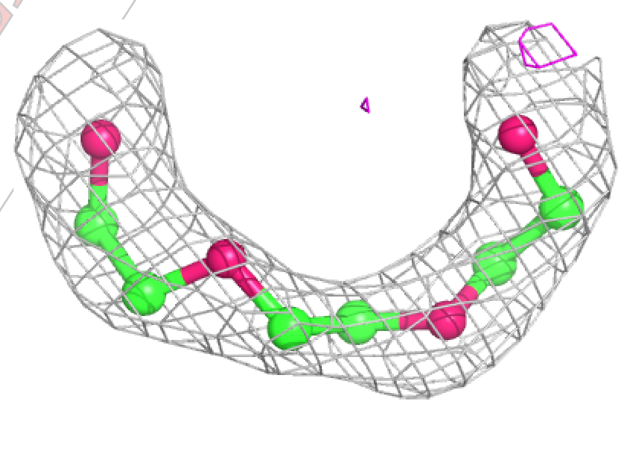

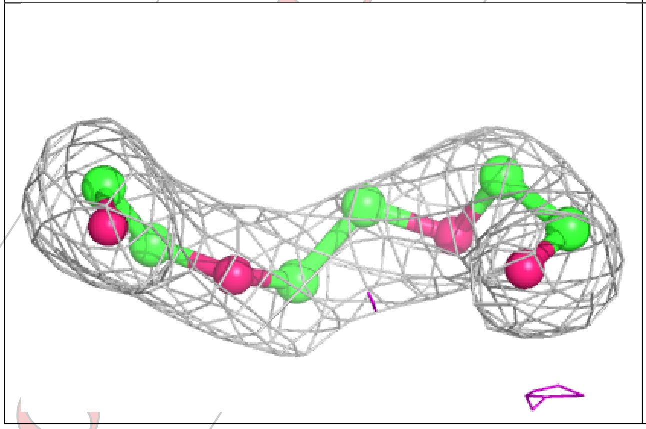

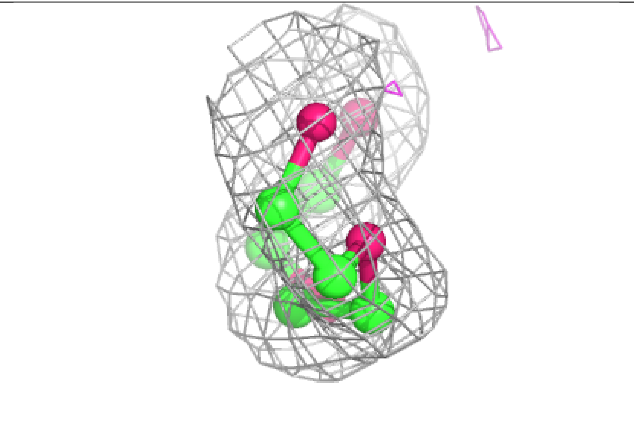

Electron density around OLC A 403:

$2mF_o$-DF_c (at 0.7 rmsd) in gray
mF_o-DF_c (at 3 rmsd) in purple (negative)
and green (positive)

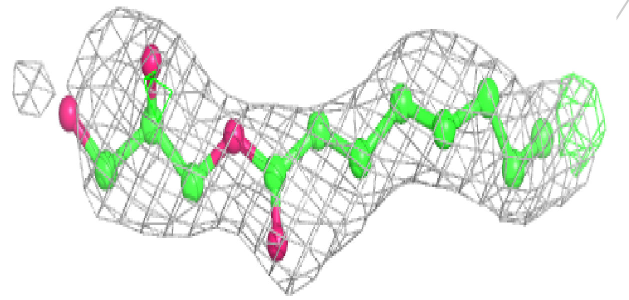

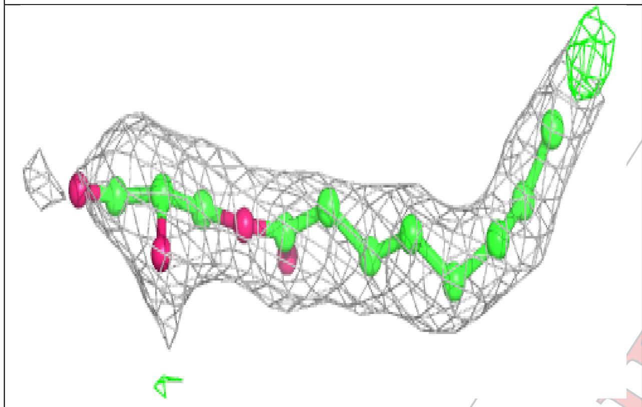

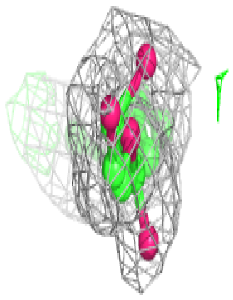

Electron density around OLC A 405:

$2mF_o$-DF_c (at 0.7 rmsd) in gray
mF_o-DF_c (at 3 rmsd) in purple (negative)
and green (positive)

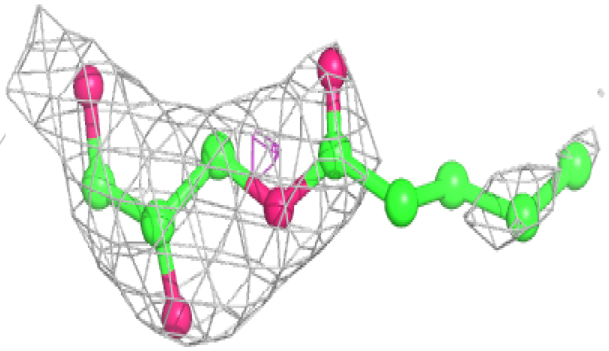

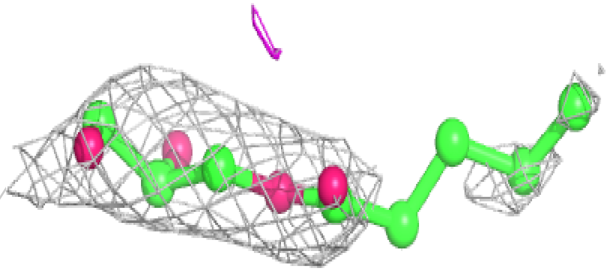

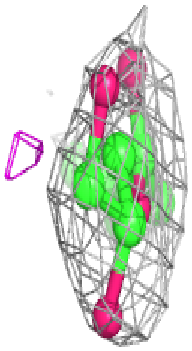

Electron density around OLC A 409:

2mF$_o$-DF$_c$ (at 0.7 rmsd) in gray
mF$_o$-DF$_c$ (at 3 rmsd) in purple (negative)
and green (positive)

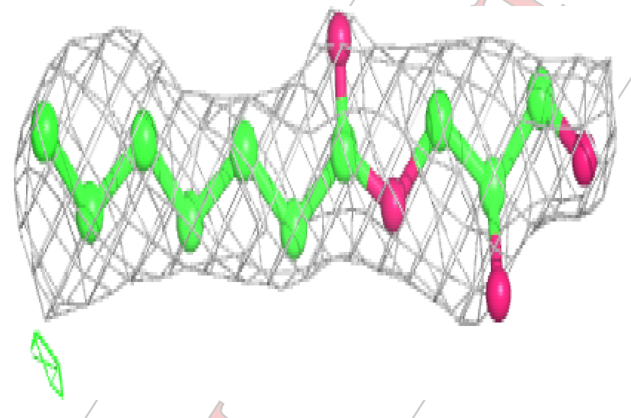

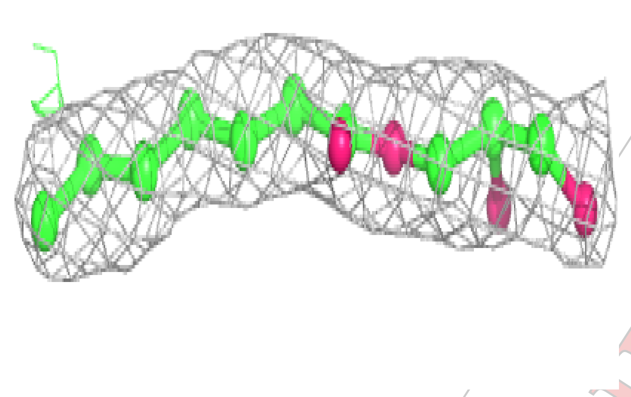

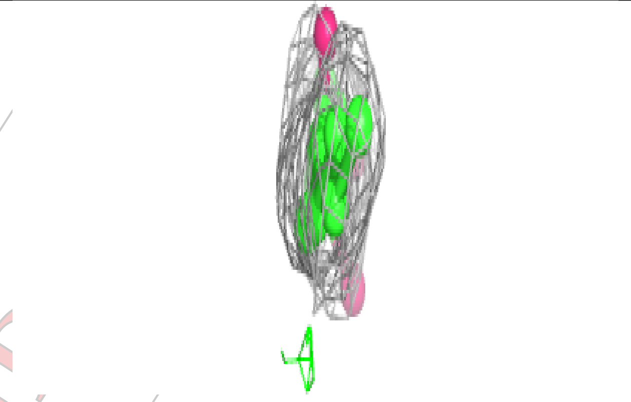

Electron density around OLC A 408:

2mF$_o$-DF$_c$ (at 0.7 rmsd) in gray
mF$_o$-DF$_c$ (at 3 rmsd) in purple (negative)
and green (positive)

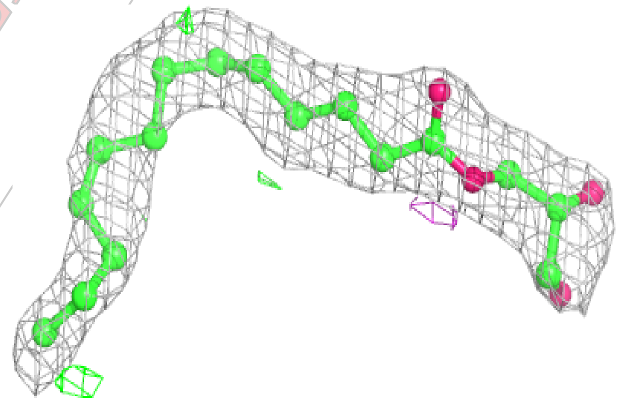

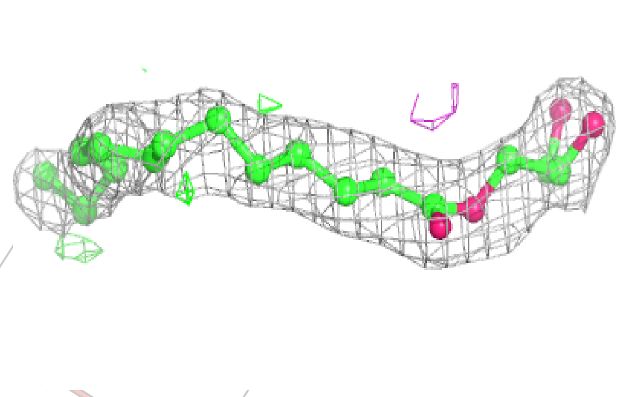

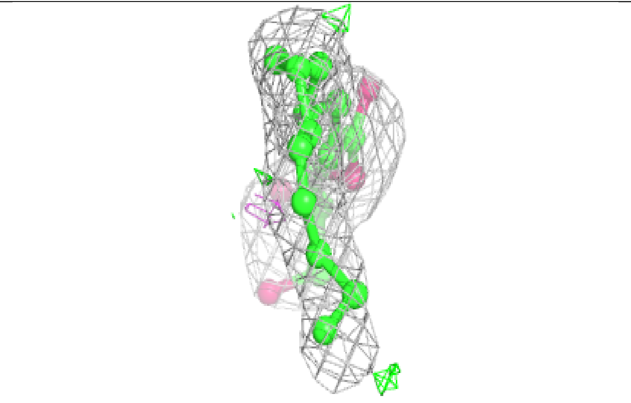

Electron density around 2PE A 415:

2mF$_o$-DF$_c$ (at 0.7 rmsd) in gray
mF$_o$-DF$_c$ (at 3 rmsd) in purple (negative)
and green (positive)

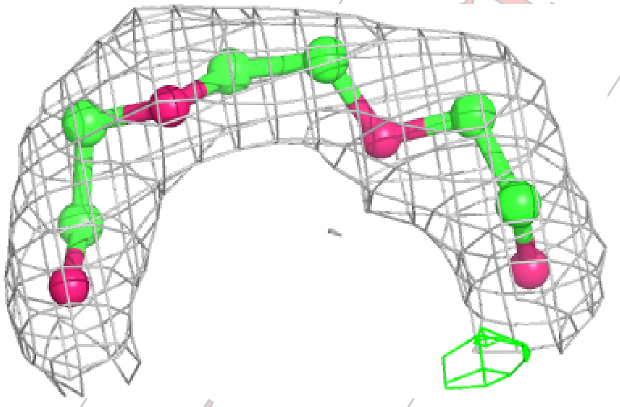

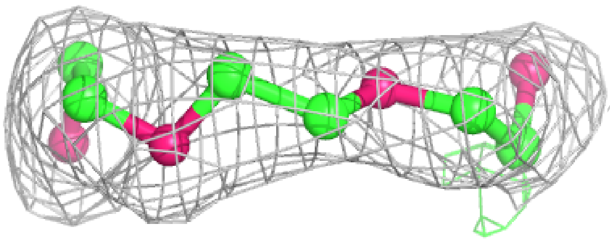

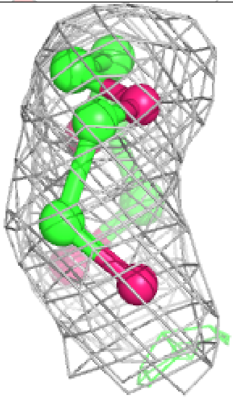

Electron density around OLC A 407:

2mF$_o$-DF$_c$ (at 0.7 rmsd) in gray
mF$_o$-DF$_c$ (at 3 rmsd) in purple (negative)
and green (positive)

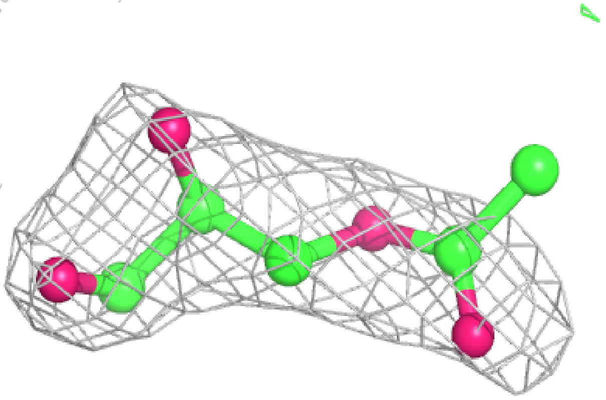

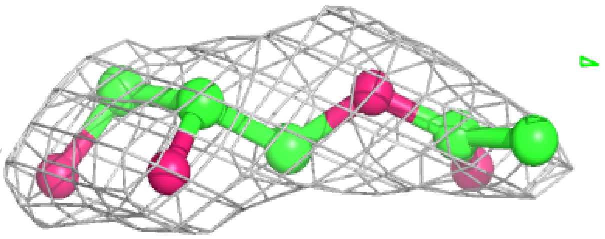

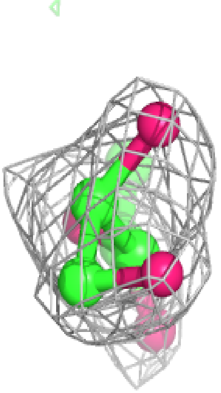

Electron density around OLC A 402:

$2mF_o$-DF_c (at 0.7 rmsd) in gray
mF_o-DF_c (at 3 rmsd) in purple (negative)
and green (positive)

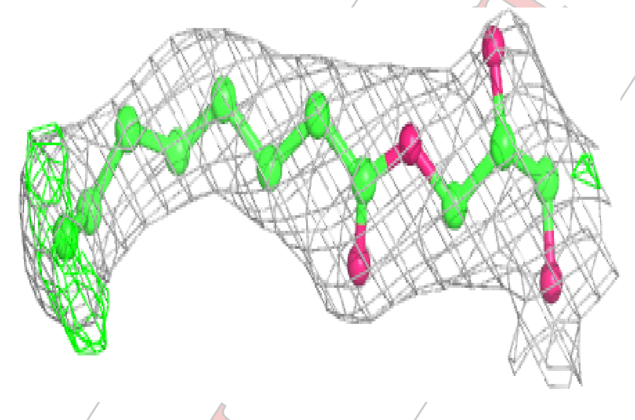

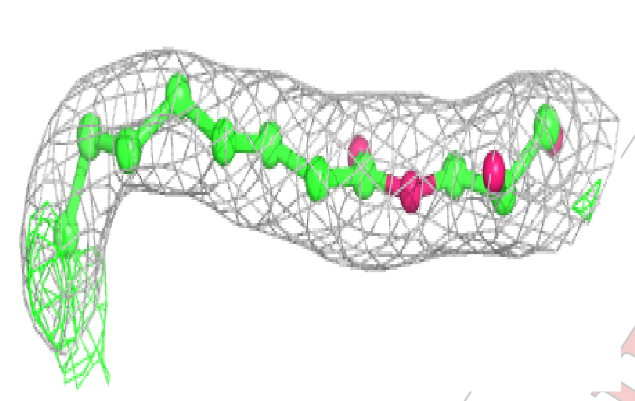

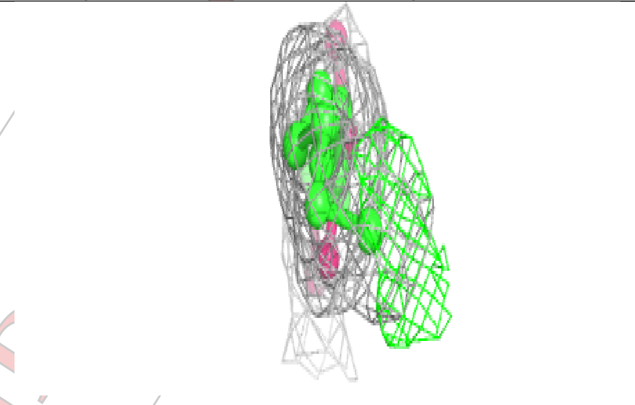

Electron density around OLC A 413:

$2mF_o$-DF_c (at 0.7 rmsd) in gray
mF_o-DF_c (at 3 rmsd) in purple (negative)
and green (positive)

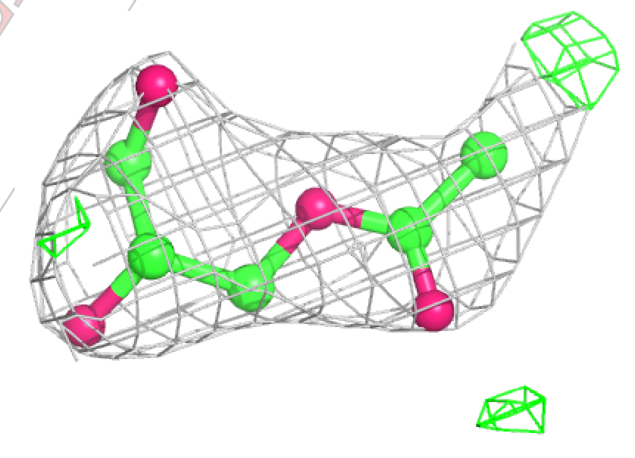

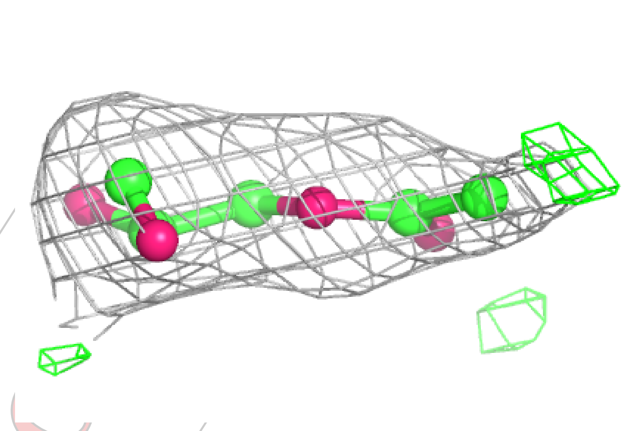

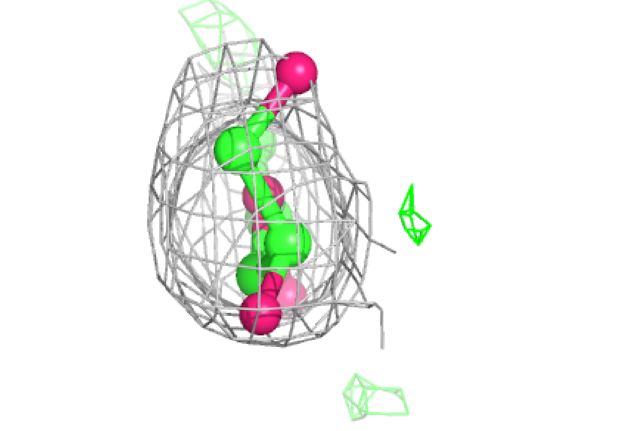

Electron density around OLC A 404:

$2mF_o$-DF_c (at 0.7 rmsd) in gray
mF_o-DF_c (at 3 rmsd) in purple (negative)
and green (positive)

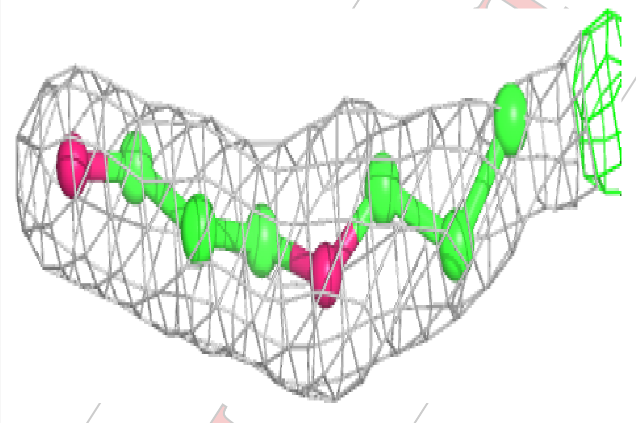

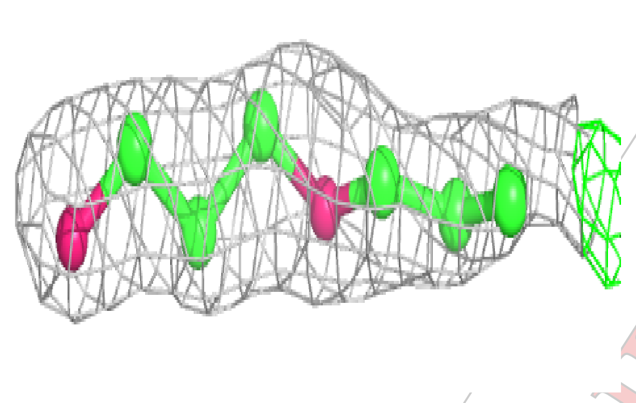

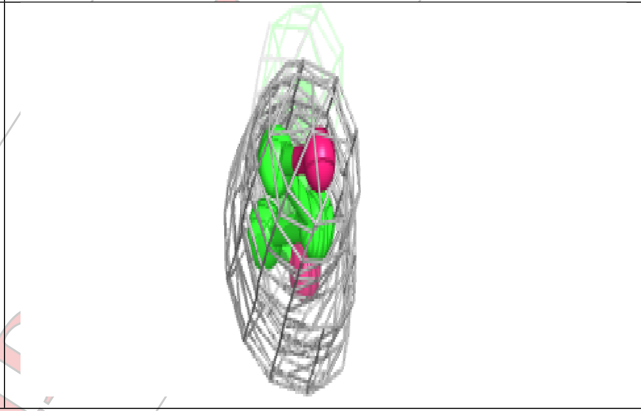

Electron density around 5MC A 401:

$2mF_o$-DF_c (at 0.7 rmsd) in gray
mF_o-DF_c (at 3 rmsd) in purple (negative)
and green (positive)

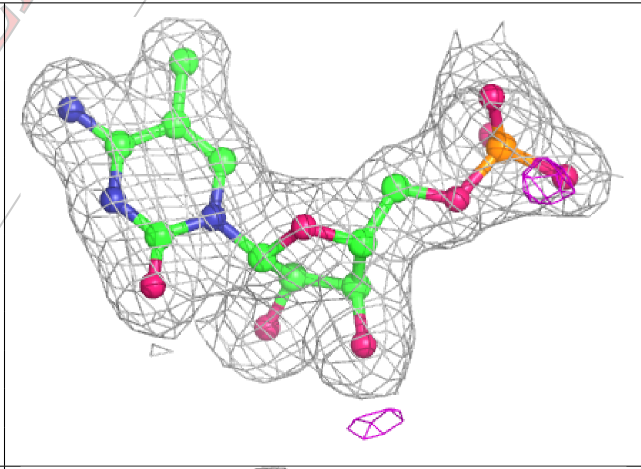

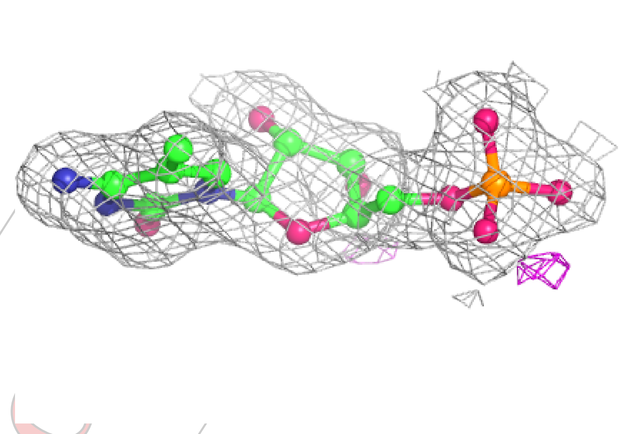

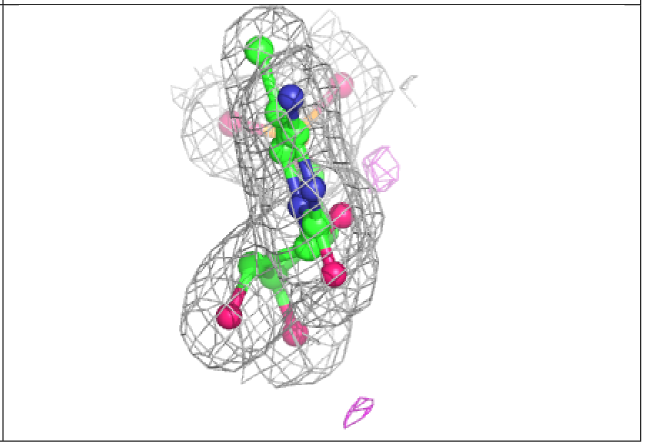

6.5 Other polymers (i)

There are no such residues in this entry.

CONFIDENTIAL VALIDATION REPORT
